# Scaling marine fish movement behavior from individuals to populations

**DOI:** 10.1002/ece3.4223

**Published:** 2018-06-25

**Authors:** Christopher A. Griffiths, Toby A. Patterson, Julia L. Blanchard, David A. Righton, Serena R. Wright, Jon W. Pitchford, Paul G. Blackwell

**Affiliations:** ^1^ School of Mathematics and Statistics University of Sheffield Sheffield UK; ^2^ Institute for Marine and Antarctic Studies University of Tasmania Hobart TAS Australia; ^3^ Centre for Environment Fisheries and Aquaculture Science Lowestoft Laboratory Lowestoft UK; ^4^ CSIRO Marine and Atmospheric Research Hobart TAS Australia; ^5^ Department of Biology University of York York UK

**Keywords:** Atlantic cod, data storage tags, European plaice, hidden Markov modeling, movement behavior, population‐level patterns, priors

## Abstract

Understanding how, where, and when animals move is a central problem in marine ecology and conservation. Key to improving our knowledge about what drives animal movement is the rising deployment of telemetry devices on a range of free‐roaming species. An increasingly popular way of gaining meaningful inference from an animal's recorded movements is the application of hidden Markov models (HMMs), which allow for the identification of latent behavioral states in the movement paths of individuals. However, the use of HMMs to explore the population‐level consequences of movement is often limited by model complexity and insufficient sample sizes. Here, we introduce an alternative approach to current practices and provide evidence of how the inclusion of prior information in model structure can simplify the application of HMMs to multiple animal movement paths with two clear benefits: (a) consistent state allocation and (b) increases in effective sample size. To demonstrate the utility of our approach, we apply HMMs and adapted HMMs to over 100 multivariate movement paths consisting of conditionally dependent daily horizontal and vertical movements in two species of demersal fish: Atlantic cod (*Gadus morhua*;* n* = 46) and European plaice (*Pleuronectes platessa*;* n* = 61). We identify latent states corresponding to two main underlying behaviors: resident and migrating. As our analysis considers a relatively large sample size and states are allocated consistently, we use collective model output to investigate state‐dependent spatiotemporal trends at the individual and population levels. In particular, we show how both species shift their movement behaviors on a seasonal basis and demonstrate population space use patterns that are consistent with previous individual‐level studies. Tagging studies are increasingly being used to inform stock assessment models, spatial management strategies, and monitoring of marine fish populations. Our approach provides a promising way of adding value to tagging studies because inferences about movement behavior can be gained from a larger proportion of datasets, making tagging studies more relevant to management and more cost‐effective.

## 1 INTRODUCTION

The spatial management of the marine world requires in‐depth information about how animals move, when they move, and where they move to. Key to increasing our understanding of species space use, movement patterns, and how individuals interact with the environment they inhabit is the rising deployment of small and reliable data loggers and transmitters on free‐roaming marine animals (Costa, Breed, & Robinson, 2012; Hays et al., 2016; Hussey et al., 2015). Capable of recording a range of movement metrics, including horizontal and vertical movement alongside basic environmental information such as water temperature, salinity, and ambient daylight, these devices have revolutionized our understanding of fundamental ecology (Hussey et al., 2015), documented oceanwide dispersal events (Block et al., 2011), highlighted areas that are essential for species survival (Raymond et al., 2015), and even allowed us to test the effectiveness of current conservation policies (Pittman et al., 2014; Scott et al., 2012).

One of the main motivations for animal‐borne telemetry studies is that by understanding individual movement behavior, we might infer the population‐, species‐ and community‐level consequences of movement (Block et al., 2011; Hindell et al., 2016; Raymond et al., 2015; Wakefield et al., 2011). This is especially true in marine systems, as individual observations provide our only insight into the otherwise unobservable. Achieving this scaling of inference from individual movement patterns to population dynamics requires two important components. The first is an adequate sample size (number of individuals) to address the ecological question of interest (Hebblewhite & Haydon, 2010) and second, a statistical means by which we gain meaningful inference at the individual and population level from a finite sample of individuals (Jonsen, 2016; Langrock et al., 2012; McClintock, Russell, Matthiopoulos, & King, 2013).

The issue of sample size has been extensively discussed, especially when considering how movement studies can inform marine conservation and spatial management (Hebblewhite & Haydon, 2010; McGowan et al., 2017; Nguyen et al., 2017; Ogburn et al., 2017). Tags can be expensive (McGowan et al., 2017), are liable to occasional failure or loss, and often result in individual pathways that are data‐poor or have a low number of observations. As a result, meeting the minimum sample size of 20 +  individuals when making simple statistical comparisons between populations is uncommon (Hebblewhite & Haydon, 2010), with even greater numbers needed when testing for the effects of age, sex, and species identity (Lindberg & Walker, 2007). In the absence of a collaborative effort across multiple institutions (Block et al., 2011; Hindell et al., 2016), a significant increase in funding or a community‐wide shift to data sharing (e.g., via online data repositories like Movebank ‐ Kranstauber et al., 2011); it would appear that the most viable route toward robust population‐level inferences is approaches that make the most of the tagging data we already have.

Among the many methodological developments that utilize movement data to answer ecological questions, hidden Markov models (HMMs) and hidden semi‐Markov models have taken center stage (e.g. DeRuiter et al., 2016; McKellar, Langrock, Walters, & Kesler, 2015; Michelot, Langrock, & Patterson, 2016; Towner et al., 2016). Favored because they match our initiative understanding that movement is governed by switches in an animal's motivation (Patterson et al., 2017), HMMs provide a computationally efficient means of objectively classifying movement into discrete states, with different statistical properties, indicating differences in underlying behavior (Langrock et al., 2012).

HMMs have been fitted to multiple individual pathways simultaneously in both the frequentist (Langrock et al., 2012; McKellar et al., 2015) and Bayesian statistical paradigms (Jonsen, 2016; McClintock et al., 2013). However, these approaches are typically implemented by specialist statisticians and require the coupling of HMM and hierarchical structures, producing a hierarchical Hidden Markov model (HHMM). The alternative is the use of HMMs or other state‐space approaches that fit on an individual by individual basis (Jonsen, Myers, & James, 2007; Michelot et al., 2017). This latter, more frequently used approach has its advantages, the most notable being an ease of use for statisticians and biologists alike. Fitting per individual also has its disadvantages. The first is that it requires individual movement paths that are suitably data‐rich to achieve model convergence, imposing even stricter restrictions on sample size. The second is a distinct lack of any formal process by which state one in animal A is ensured consistency with state one in animal B. This lack of consistency means that estimated parameters can readily inform individual‐level movement studies but will result in tricky interspecific and intraspecific comparisons, limiting a researcher's ability to ask *post hoc* population‐level questions of their data.

Our objective is to introduce an alternative framework that uses HMMs to overcome the described limitations of individually fitted HMMs while maintaining their heralded ease of use advantages. Our approach combines an *N*‐state HMM and several hierarchical structures but bypasses the need to integrate over the random effects (as in HHMMs; Langrock et al., 2012) by using information we gain from our data‐rich pathways as *a priori* approximations of each states movement parameters. Doing so not only allows us to achieve coherent individual‐ and population‐level state classification, but also ensures that we maximize our sample size by gaining meaningful inference from our data‐poor and data‐rich movement paths.

To illustrate our approach, we apply it to a real ecological problem—quantifying seasonal space use patterns in Atlantic cod (*Gadus morhua*) and European plaice (*Pleuronectes platessa*) in the North Sea and English Channel. Both Atlantic cod and European plaice have significant commercial and conservation value and as a result have been the subject of several long‐term tagging programs (Hobson, Righton, Metcalfe, & Hays, 2007, 2009; Hunter, Metcalfe, Arnold, & Reynolds, 2004; Hunter, Metcalfe, O'Brien, Arnold, & Reynolds, 2004; Righton, Metcalfe, & Connolly, 2001). Drawing on this, the rest of this paper considers a case study of 107 individual bivariate movement paths, many of which (*n* = 73) have limited observations and/or lack clear biological signals. Our findings demonstrate clear spatiotemporal patterns in the movement behavior of either species that are consistent with individual‐level studies (Hobson et al., 2007, 2009; Hunter, Metcalfe, Arnold, et al., 2004; Hunter, Metcalfe, O'Brien, et al., 2004; Neat et al., 2014). Furthermore, by analyzing a relatively large dataset, we provide a unique insight into how differing substocks of cod and plaice shift their behavior on a seasonal basis, with clear consequences for fisheries management and conservation.

## 2 MATERIALS AND METHODS

### 2.1 Case study data

**Figure 1:**
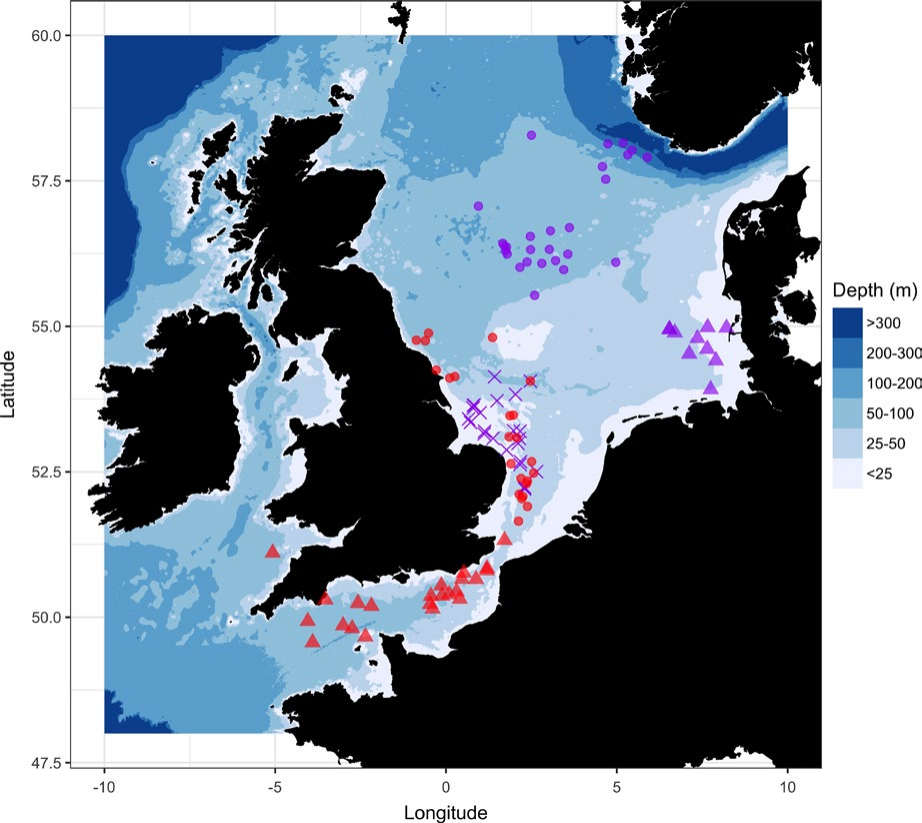
Release locations of all tagged fish. Atlantic cod, *Gadus morhua* (*n* = 46) are shown in red, fish are either separated into the English Channel substock (triangles, *n* = 23) or the southern North Sea substock (circles, *n* = 23). European plaice, *Pleuronectes platessa* (*n* = 61) are shown in purple, fish are grouped into three substocks: Central North Sea (circles, *n* = 27), German Bight (triangles, *n* = 10), and Southern North Sea (crosses, *n* = 24)

Movement paths were taken directly from the deployment of data storage tags (DSTs) on free‐roaming fish in the North Sea or English Channel. The dataset includes 107 individuals from two species of European demersal fish: Atlantic cod (*n* = 46) and European plaice (*n* = 61). All fish were tagged and released between December 1996 and June 2011. Fish were broadly separated into substocks based on release location (see Figure 1) and displayed considerable variation in movement path duration (Supporting information: Table S1).

Each DST was programmed to record depth (m) at 10‐min intervals for the duration of deployment. The first 2 weeks and the last day of every time series were excluded to remove any erroneous or irregular measurements associated with release and recapture events as per Hobson et al. (2007). For details of tag type, fish catchment, tag implantation and measurement accuracy see Righton et al. (2010; *Gadus morhua*) or Hunter, Metcalfe, Arnold, et al. (2004; *Pleuronectes platessa*).

Each movement path is a bivariate time series of horizontal and vertical movement per day. Net vertical movement (m/day) of each fish was taken directly from the raw DST data by calculating the absolute difference between corresponding 10‐min depth measurements and summing the values for each day at liberty. Horizontal movement (m/day), in comparison, was inferred indirectly from the depth data in a two‐step approach. First, daily geolocation estimates were produced via a Fokker–Planck‐based method that combines Metcalfe and Arnold's (1997) tidal location method and a Bayesian state‐space model (see Pedersen, Righton, Thygesen, Andersen, & Madsen, 2008 for model details). The straightline distance between daily geographic estimates (commonly referred to as “step‐length”) was then calculated using the Great Circle equation. Both vertical (*v*) and horizontal (*h*) movement metrics were log (natural log) transformed prior to model implementation. Only time series that were longer than 40+ days and had complete depth recordings were used in this study. For descriptions of horizontal and vertical movement in Atlantic cod and European plaice see Hunter, Metcalfe, Arnold et al. (2004), Hunter, Metcalfe, O'Brien et al. (2004) and Hobson et al. (2007, 2009).

### 2.2 The model

Previous individual‐level studies demonstrate that Atlantic cod and European plaice display periods of high activity while in the water column punctuated by periods of relatively low activity while on the seabed (Metcalfe, Hunter, & Buckley, 2006; Righton et al., 2010). Thus, we consider a discrete 2‐state HMM. We label state one as “resident” (*R*), representing periods of time with low movement rates. We label state two as “migrating” (*M*), representing a much more active phase where movement rates in the horizontal and vertical dimension are greatly increased. As in all attempts to infer behavior from movement observations, state labels must be interpreted with care as they provide simplified proxies of unobserved behavioral modes, not direct equivalents (Patterson et al., 2017).

For a movement path of length T, it is assumed that an underlying, nonobserved state sequence *S*_1_, …, *S*_T_, taking values in {*R*,* M*} describes the persistence within and stochastic switching between states. The time‐varying evolution of this state process takes the form of a (first‐order) Markov chain, with transition probability matrix Γ

(1) $$\Gamma =\left(\begin{array}{c} \gamma_{R\to R}\\ \gamma_{M\to R} \end{array}\begin{array}{c} \gamma_{R\to M}\\ \gamma_{M\to M} \end{array}\right)$$

(2) $$\gamma_{j\to k}=\mathrm{Pr}(S_{t+1}=k\left|S_{t}=j\right)$$

andfor any *j*,* k* in {*R*,* M*}. Given a state *j* at time *t* the observation *x*_*t*_ is assumed to be drawn from a multivariate normal distribution (MVN):

(3) $$x_{t}∼\text{MVN}\left(\mu_{j},\Sigma_{j}\right)$$

with

(4) $$\mu_{j}=\left(\begin{array}{c} \mu_{\mathrm{jH}}\\ \mu_{j}V \end{array}\right)$$

and

(5) $$\Sigma_{j}=\left(\begin{array}{c} \sigma_{\mathrm{jH}}^{2}\\ \rho_{j}\sigma_{\mathrm{jH}}\sigma_{\mathrm{jV}} \end{array}\begin{array}{c} \rho_{j}\sigma_{\mathrm{jH}}\sigma_{\mathrm{jV}}\\ \sigma_{\mathrm{jV}}^{2} \end{array}\right)$$

and *H* and *V* represent movements made in the horizontal and vertical dimension, respectively. Thus, the complete‐data likelihood given a state sequence *S*_1_, …, *S*_T_ is

(6) $$\omega_{S_{1}}\phi_{S_{1}}\left(x_{1}\right)\gamma_{S_{1}\to S_{2}}\phi_{S_{2}}\left(x_{2}\right)\ldots \gamma_{S_{T-1}\to S_{T}}\phi_{S_{T}}\left(x_{T}\right)$$

where the row vector ω is the Markov chain initial state probability (which we assume to be uniform at *t *=* *1) and $\phi_{j}$ refers to the multivariate normal density stated in equation (3). We allow distinct parameters for each fish, indexed by *i *=* *1, …, 107, and write these as $\Gamma^{i}$, $\mu_{j}^{i}$ and $\Sigma_{j}^{i}$.

In practice, standard HMM algorithms allow us to calculate the actual likelihood, when the states are unobserved, very efficiently by integrating over all possible state sequences using the forward algorithm (Zucchini, MacDonald, & Langrock, 2016). Framing the model in this way enables us to conduct parameter estimation using a Bayesian approach, by numerically maximizing the posterior density. The classification probability of each state at *t* is then determined using the backward smoothing algorithm (Zucchini et al., 2016). More details for how the efficient HMM machinery can be used to conduct statistical inference are given in Zucchini et al. (2016), for the particular case of animal movement modeling see Patterson et al. (2017). For our case study, we used the R optimization routine *optim* to numerically maximize the log posterior density. State allocation is carried out by selecting the most likely state at each time point separately.

Periods of relative inactivity (low *h* and *v* movement rates) can persist for 3‐5 months in either species (Metcalfe et al., 2006; Righton et al., 2010). To accommodate this persistence within state, we have imposed a prior penalty term on the transition probabilities, such that

(7) $$\gamma_{11}∼\beta \left(\alpha ,\beta \right)$$

and

(8) $$\gamma_{22}∼\beta \left(\alpha ,\beta \right)$$

where α = 99 and β = 1. This prior, termed hereafter as the transition probability prior, is designed to ensure that states *R* and *M* correspond to strong seasonal shifts in movement behavior and not day‐to‐day fluctuations.

### 2.3 Classifying fish movements

We apply the model described in section 2.2. to all 107 individual movement paths, such that each fish gets its own parameter set. Each parameter set consists of 12 estimated parameters, two transition probabilities and 2 sets of 5 parameters describing the mean ($\mu_{j}$) and covariance ($\Sigma_{j}$) of each state. A total number of 24,624 days (Atlantic cod = 9290 days; European plaice = 15,334 days) were considered. As expected, the resulting state sequences are predominately made up of two clearly defined behavioral modes – one more active and one less active (see Supporting information: Figures S1 and S2 for example output). However, the parameters describing the numerical structure of these modes showed great variation among fish, with no clear consistency. Moreover, a handful of movement paths failed to achieve model convergence, as an upper threshold of observations is needed for robust parameter estimation (Patterson, Basson, Bravington, & Gunn, 2009).

**Figure 2:**
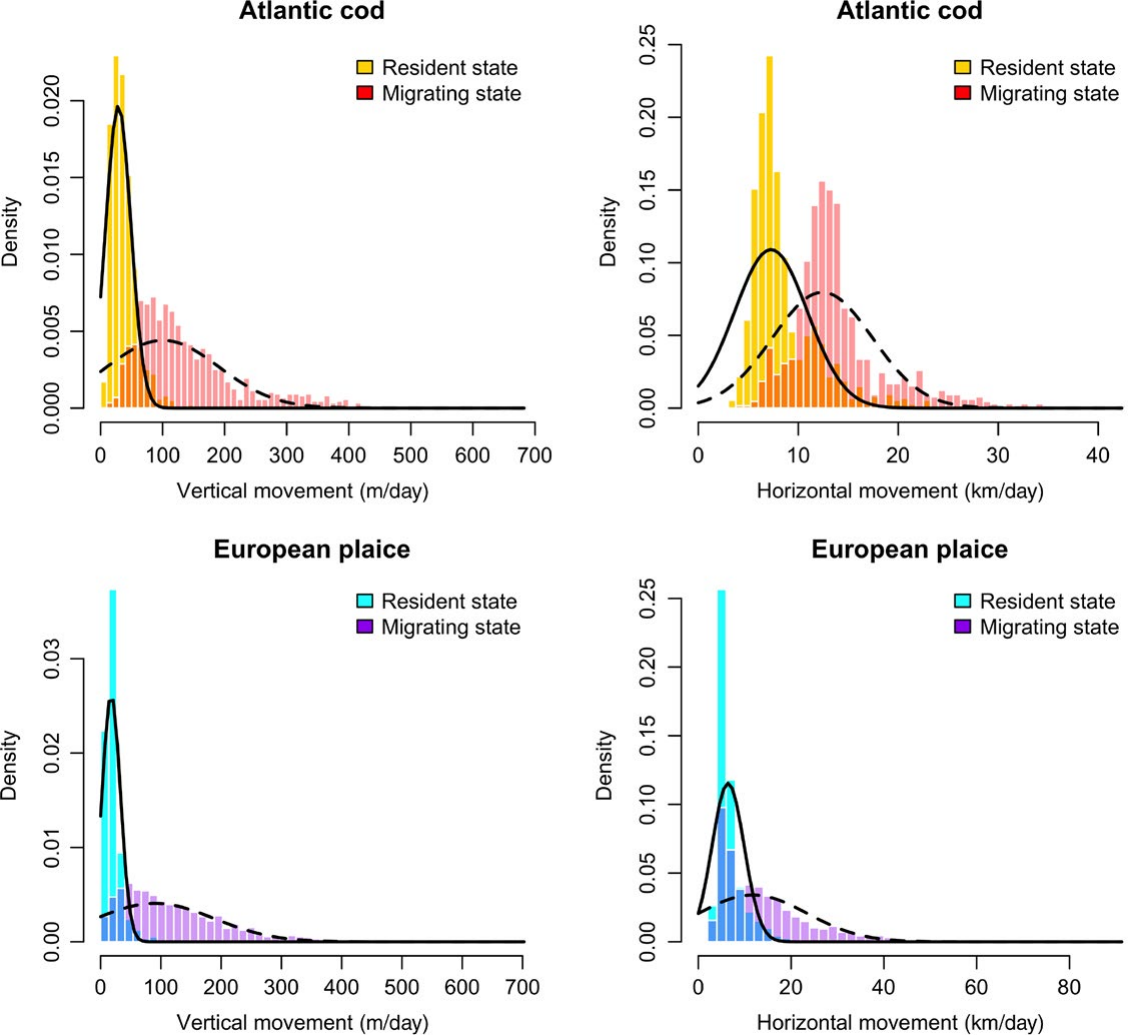
Estimated state‐dependent distributions (bars) for vertical (left) and horizontal (right) movements of all 34‐selected fish. Black lines illustrate the movement parameter prior distributions $N\left(m,\delta \right)$ that were constructed based on collective model output. Prior distributions are state (resident, solid line; migratory, dashed line)‐, species (Atlantic cod, top; European plaice, bottom)‐, and dimension (horizontal or vertical)‐specific

To avoid the wasteful removal of valuable data or a tedious *post hoc* description of the individual variation that exists in the HMMs output, we adopted an alternative approach. Based on the selection criteria outlined in Supporting information: Figure S3, we select model output from 34 fish (Atlantic cod, *n* = 11; European plaice, *n* = 23) spread evenly across the five substocks (Supporting information: Table  S2). We then calculate summary statistics (means *m* and variances δ) that describe the numerical structure of the two states (Supporting information: Figure S4). These summary statistics are used to construct Gaussian distributions (Figure 2), $N\left(m,\delta \right)$ where *m* and δ are dimension (*h* or *v*) *d*, state *j* and species specific given the selected sample. These informative distributions (4 per species), termed hereafter as priors on the model's movement parameters, are then introduced directly into the HMMs likelihood function, such that Equation (6) is multiplied by

(9) $$\prod_{j}\prod_{d}\phi \left(\mu_{\mathrm{jd}}|m_{\mathrm{jd}},\delta_{\mathrm{jd}}\right)$$

where ϕ( · | *m*, *δ*) is the Gaussian density with mean *m* and variance δ. Thus, our informative priors act to constrain the mean parameters of each state during the classification process.

This adapted approach is applied to the classification of the remaining 73 individual pathways (Atlantic cod, *n* = 35; European plaice, *n* = 38), outputting state sequences that comprise comparable states across all fish. This enables *post hoc* comparisons to be made at the individual and population level with relative ease. For an example of how prior inclusion influences the classification process see Supporting information: Figure S5. Furthermore, demonstrations of how comparable states are across multiple fish (Supporting information: Figure S6) and differences between model fit for one of the data‐poor movement paths are provided (Supporting information: Figure S7).

All HMMs were coded and implemented in R (R Development Core Team, 2016; see Supporting Information document 2 for example code). All plots were generated using the *ggplot2* (Wickham, 2009) and *ggmap* (Kahle & Wickham, 2013) packages in R (R Development Core Team, 2016). Bathymetric data was sampled from the General Bathymetric Chart of the Oceans online repository (GEBCO 2017, http://www.gebco.net), which is a global topographic dataset with a one‐minute (1’) spatial resolution.

### 2.4 Prior sensitivity analysis

When imposing prior distributions in statistical models it is always important to test what influence those priors have on the models’ predictions, in our case the model's estimated state sequences. To test the sensitivity of our model to changes in the transition probability prior we varied the α and β values that characterize the priors’ beta distribution and reran the HMM for all 34 “selected” fish. In test 1 (α = 49.5, β = 0.5) we still expect a behavioral switch to occur at an order of every 100 days. However, we approximately double our prior's variance. In test 2 (α = 49, β = 1) the expected rate of switching is halved.

To test the model's sensitivity to changes in the movement parameter priors, we varied the variances (δs) that describe the spread of each state and reran the adapted HMM for 10 randomly selected fish from each species. In test A, we increased all δ values by 10%, reflecting a prior expectation of greater variability between the parameters of individual fish, and in test B we decreased all δ values by 10%, reflecting an expectation of reduced variability. During all reruns of the adapted HMM (Test A and Test B) the state transition prior is kept constant, therefore ensuring that any change in state is a direct consequence of the changes to the model's movement parameter prior.

### 2.5 Univariate modeling

To assess the advantages of using bivariate responses, we also carried out an analysis using a univariate observation model, considering only movements made in the horizontal dimension. The same model for transition probabilities is used as described above. We apply this approach to the 34 fish (Atlantic cod, *n* = 11; European plaice, *n* = 23) previously characterized as data‐rich movement paths.Reported comparisons reflect the percentage change, if any, in the resultant state sequences for each individual fish.

### 2.6 Inferring population patterns

As population dynamics emerge as the sum of the individuals that comprise the population, we used individual movement behaviors to explore spatiotemporal patterns. Annual temporal patterns of movement behavior were calculated for each species in two ways. First, the daily individual probabilities of each fish being in each state were averaged across all individuals and over each week of the year. Second, the proportion of fish classified to each state was calculated by averaging the daily number of fish in each state and smoothing it, again to the weekly time step. Week refers to weeks of the year, starting on the 1st January and ending on the 31st December and is independent of year.

Patterns of space use while in either state were quantified using utilization distributions (Kie et al., 2010; Womble & Gende, 2013; Worton, 1989). For each species and substock, utilization distributions were calculated by pooling all daily horizontal geolocations for specified time periods and spatially binning them into 5 km^2^ grid cells (Maxwell et al., 2011; Womble & Gende, 2013). Specified time periods were state‐dependent and based on a weekly averaged probability of observing a given state across all individuals exceeding 0.5. Successive weeks classified to the same behavioral state were then grouped. In Atlantic cod this meant locations that were classified to a resident state between June – October and locations classified to a migrating state between November and May were used. In European plaice locations classified to a resident state between April and September and locations classified to a migrating state between October and March were used.

## 3 RESULTS

### 3.1 Individual fish movement

**Figure 3:**
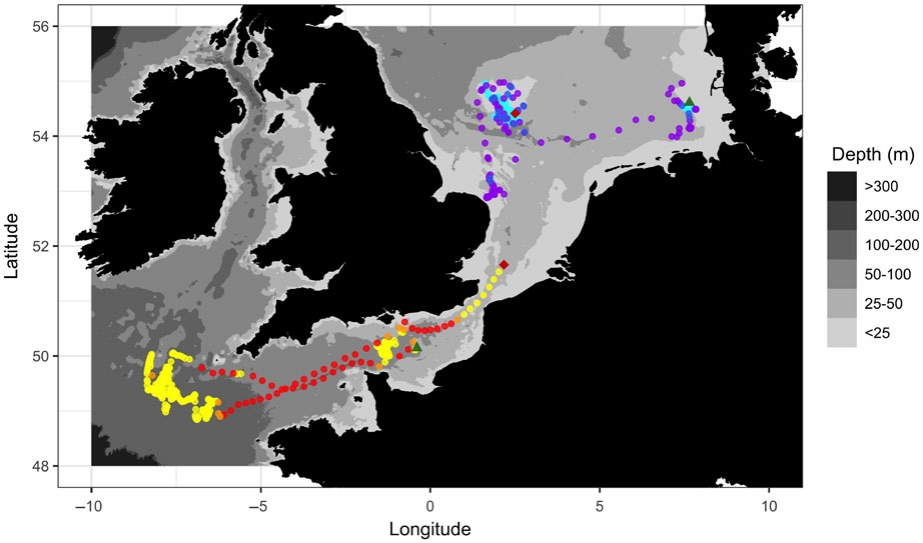
State‐dependent movement behavior of two individual fish. Shown in a color scale from red to yellow is the movement behavior of one Atlantic cod tagged on the March 25, 2005 (duration = 300 days). Red points represent a migrating state, yellow a resident state, and those points shown in orange illustrate times when the model was uncertain of state classification (i.e., the daily probability of state classification was <0.85). Shown in a scale from purple to cyan is the movement behavior of one European plaice tagged on the November 14, 1997 (duration = 253 days). Purple points represent a migrating state, cyan a resident state, and those points shown in royal blue illustrate times when the model was uncertain of state classification. The start point and end point of each individual's movement path are shown as a green triangle and a red diamond, respectively

Mapping the posterior probability of being in a particular state indicated that individual fish from either species switch between periods of highly directed movement when in a migratory state and periods of random and highly localized movements when in the less active resident state (Figure 3). Time spent in either state and the transitions between states were shown to vary in space and time and can be linked to certain habitats. For example, cod 1186 spent 197 days (June‐November) consecutively in the resident state within the deeper waters of the Celtic Sea and only shifted into a migratory state when transiting through the English Channel. In comparison, plaice 1084 undertook long‐distance directed movements after its release in the German Bight, spending 54 days consecutively in the migrating state before switching to the resident state in the shallow waters of the Central North Sea.

The majority of individual time series had observations that shifted between resident and migratory states (*n* = 41 Atlantic cod, *n* = 60 European plaice). However, a small number of individuals (*n* = 6) persisted in a single state for the duration of their time series: one European plaice and four Atlantic cod remained in a resident state throughout, whereas the movements of one Atlantic cod were consistently classified to the migratory state. All 6 single state movement paths had short duration times (average movement path duration = 56 ± 21 days) and were released throughout the year (November–May).

### 3.2 Population patterns

**Figure 4:**
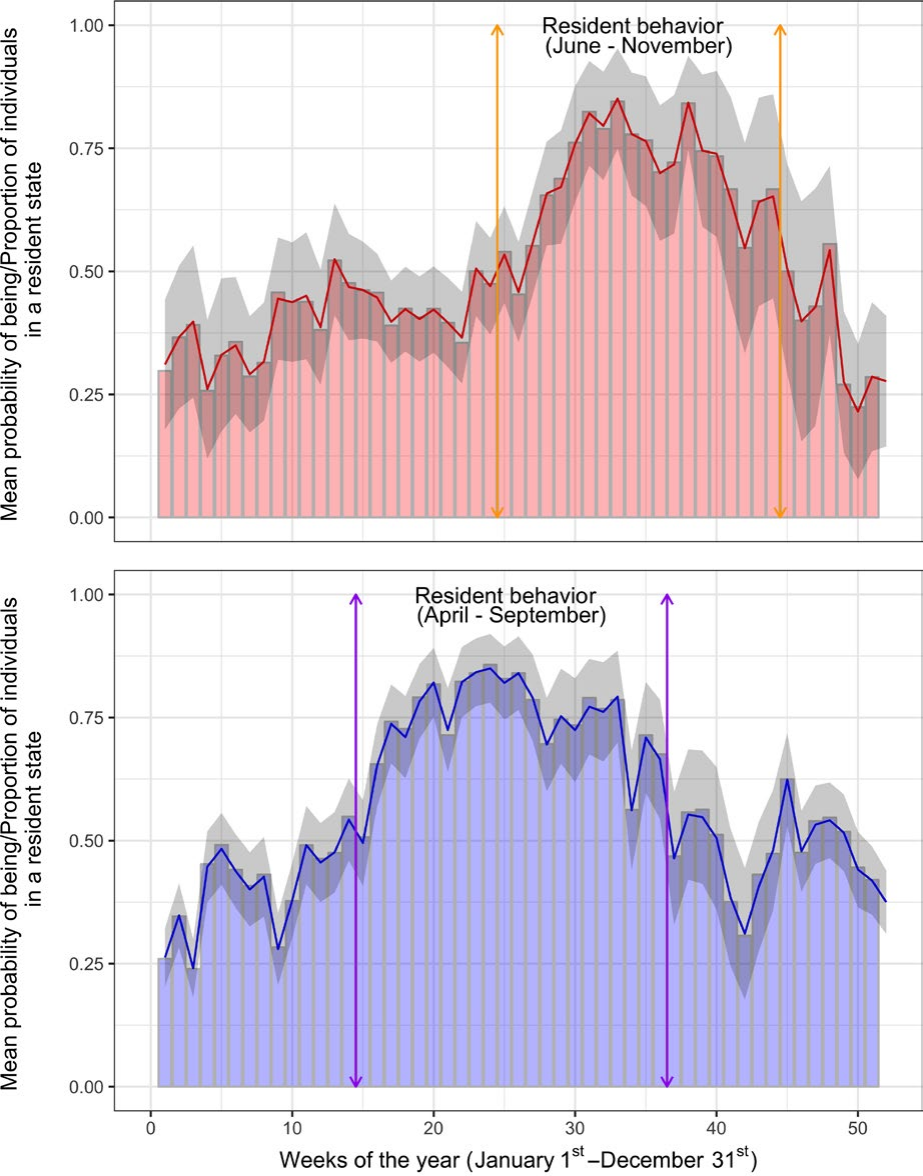
Annual temporal distributions of the resident state in Atlantic cod (red) and European plaice (blue). The plotted line in either graph illustrates the mean probability of observing a resident state (±1 *SE*—gray shading). The underlying barplots demonstrate the proportion of individual fish that are in a resident state during each week. Periods of time when the mean probability of observing a resident state is continually >0.5 are illustrated in either species

The mean probability of observing a resident state and the proportion of observations classified to a resident state varied throughout the year (Figure 4). In both species, migratory behavior dominated throughout the winter and into spring, with the onset of summer signifying a shift in movement behavior to the resident state. This shift in state occurred earlier in European plaice than in Atlantic cod, with movements of plaice having a higher probability of classification to the slower, less active resident state between late April and September, compared to June through to November in cod.

The model predicted large variation in average movement rates within each state (Table 1). Horizontal movement rates of plaice tagged and released in the Southern North Sea and German Bight were significantly lower than those tagged in the Central North Sea (resident, Student's *t* test, *p* < 0.001; migrating, Student's *t* test, *p* < 0.001). In the resident state, plaice from the Southern North Sea and German Bight moved on average 6.5 km/day horizontally and between 20.0 and 26.1 m/day vertically compared to 13.9 km/day horizontally and between 15.6 and 125.8 m/day vertically in the migratory state. In comparison, plaice tagged in the Central North Sea exhibited much higher horizontal movement rates, moving on average 12.9 and 19.5^ ^km/day in the resident and migratory states, respectively.

**Table 1:** State‐dependent movement rates (horizontal: km/day, vertical: m/day) by substock in Atlantic cod and European plaice

*Substock*	Resident state		Migrating state	
	Horizontal movement (km)	Vertical movement (m)	Horizontal movement (km)	Vertical movement (m)
Atlantic cod (*Gadus morhua)*				
Southern North Sea	9.2	31.5	13.9	158.3
English Channel	9.6	53.5	13.4	125.4
European plaice (*Pleuronectes platessa*)				
Southern North Sea	6.4	20.0	12.9	115.6
German Bight	6.6	26.1	14.9	125.8
Central North Sea	12.9	26.2	19.5	121.0

*Note*.

All values are taken from collated model output and are averaged across all individuals.

**Figure 5:**
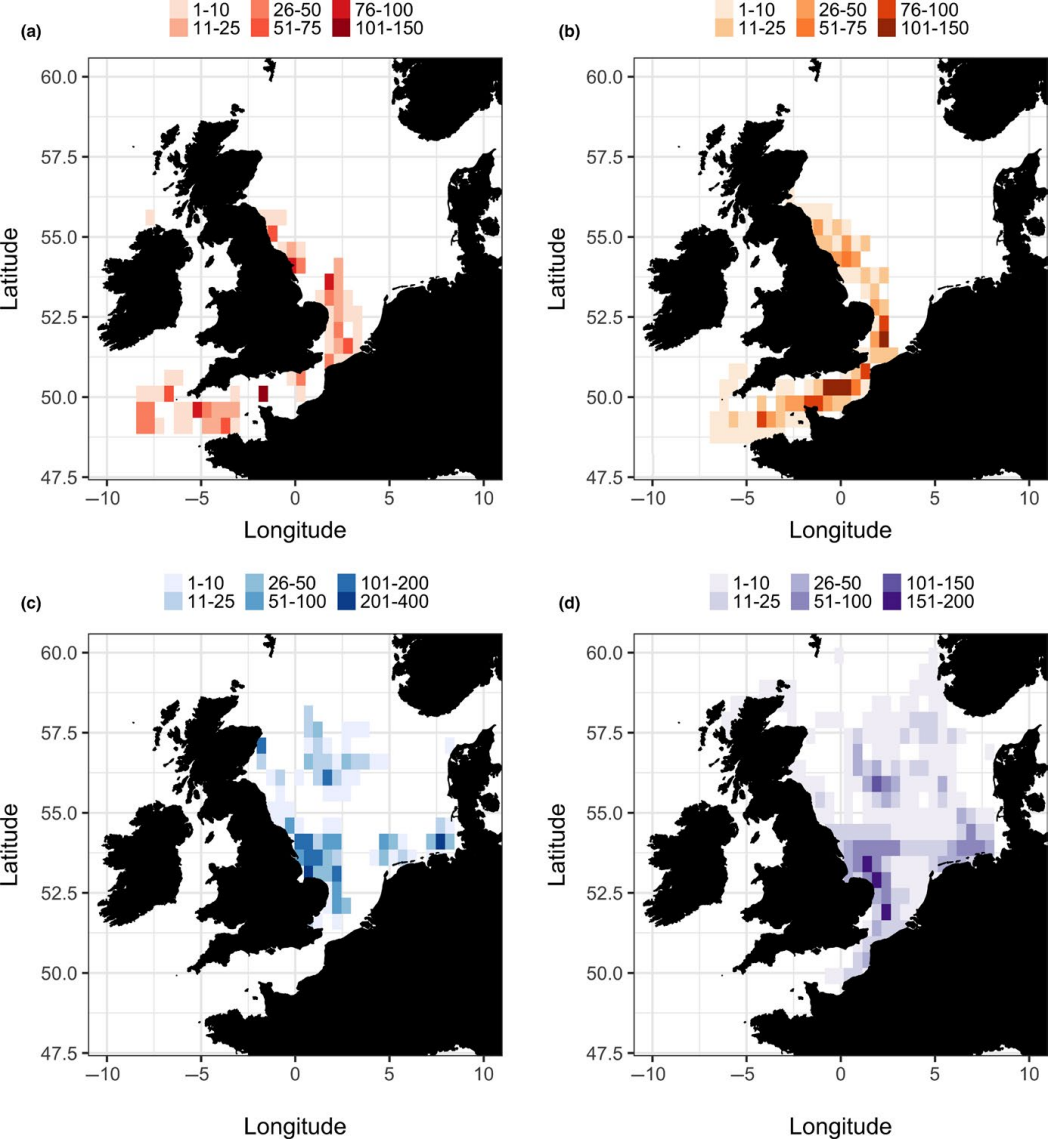
Annual state‐dependent space use patterns of Atlantic cod (a, b) and European plaice (c, d) in the North Sea and English Channel. Plots are split into periods of resident dominant (a, c) and migrating dominant (b, d), defined by a mean probability of observing a given state at a given time being >0.5. All grid cells (5 km^2^) are illustrated in a color gradient so as to illustrate the sum total number of days spent in a certain state in a given grid cell within a specified time period

Predicted spatial utilization distributions showed that migration occurred throughout the spatial domain, with no clear concentration of migratory activity in either species (Figure 5; Supporting information: Figure S8). In comparison, periods of time spent in a resident state produced clear geographic patches of space use while in certain habitats. These habitats varied with species (Figure 5) and substock (Supporting information: Figure S8), however Southern North Sea cod and plaice both aggregated in the coastal waters off the English mainland. Cod in the English Channel shift to a resident state when in the western mouth of the Channel. In the German Bight, 90% of plaice spent most of their time at liberty within the area, displaying little or no dispersal. Of those plaice tagged in the Central North Sea, 48% were estimated to be in the resident state within the Northern North Sea while a further 11 fish undertook southern migrations before shifting to a resident mode in the shallow waters of the Central North Sea.

### 3.3 Prior sensitivity analysis

Minimal change in the classification of states was found during prior sensitivity analysis (Supporting information: Table  S3). Re‐running the HMM with changes to the transition probability prior revealed an average percentage change in state across all individuals of 1.5% in cod and 1.8% in plaice. In comparison, rerunning the adapted HMM with changes to the movement parameters priors resulted in a percentage change in state that was on average <1% in cod and 2.3% in plaice. Such findings demonstrate that the precise details of these priors are not crucial, with state classifications and biologically important results being robust to fairly large changes in prior parameters.

### 3.4 Distribution of state dwell times

In an HMM, the length of time that an individual spends in one state before switching to the other necessarily follows a geometric distribution. Pooling across individuals, we find that these distributions are indeed geometric (see Supporting information: Figures S9 and S10), and so the dynamics of the fitted changes in state are consistent with the Markov nature of the model. Further model assessment is provided by residual plots in Supporting information: Figure S11 and S12.

### 3.5 Comparison to univariate modeling

State allocation was found to be different across the two tested observation models. The bivariate model resulted in state sequences that differed from the univariate model in 8.0% and 23.3% of cases in Atlantic cod and European plaice, respectively. This result confirms the need for the bivariate analysis.

## 4 DISCUSSION

One of the main objectives of animal movement studies is the scaling of inference about movement behaviors from individuals to populations (Block et al., 2011; Hays et al., 2016; Hindell et al., 2016; Raymond et al., 2015; Wakefield et al., 2011). HMMs (McKellar et al., 2015; Michelot et al., 2016; Patterson et al., 2009) or their Bayesian equivalents (Jonsen et al., 2013; McClintock et al., 2013) provide a powerful way of achieving this objective but only when movement behaviors are identified consistently across multiple individuals. Here we have achieved this consistency by “borrowing” information from a finite sample of individuals and using it to provide our model with data‐driven approximations of each state. Using this novel extension to HMM methodology, we investigated spatial and temporal shifts in movement behavior from a large sample size of bivariate movement pathways. We demonstrated where and when shifts between two ecologically meaningful states are most likely to occur and add further confidence to observations of seasonal dependence in the movements of commercially important demersal fish. Our biological findings complement and advance current understanding and highlight how our approach has significant utility in the fields of movement ecology and conservation.

Our approach to behavior classification has two major advantages. First, it enabled us to gain meaningful inference from 73 (68% of the dataset) additional movement pathways, many of which are data‐poor and would otherwise be subject to *post hoc* removal. This retention of all individual‐level information is favorable because it maximized our sample size and lends more information to our analysis. Second, our approach ensures that state labels are allocated consistently across multiple individuals, without resorting to large increases in model complexity. As a direct consequence of these two advantages, we were able to ask population‐level *post hoc* questions of our movement data and provide answers that are meaningful for conservation and spatial management.

Studies that classify behavior based on horizontal and vertical movements are rare (but see Breed, Bowen, & Leonard, 2013; Bestley, Jonsen, Hindell, Harcourt, & Gales, 2015; DeRuiter et al., 2016). Here, we have assumed that *h*_*t*_ and *v*_*t*_ are conditionally dependent given latent states, which is a novel addition to the movement ecology literature. Our reasons for doing so are linked to *a priori* information about how the species of interest alter their activity levels within an annual cycle (Hobson et al., 2009). However, we intuitively expect other species occupying three‐dimensional environments to exhibit similar degrees of coupling. For example, Bestley et al. (2015) reveal that the directed horizontal movements in multiple Antarctic pinniped species are associated with longer dive durations, whereas an inverted relationship is noted in blue whales (*Balaenoptera musculus*) with perceived shallow foraging behaviors being characterized by shallow dives and short horizontal movements (DeRuiter et al., 2016). Future studies may find similar observation models a powerful tool for investigating the dependences of horizontal and vertical movement rates (Carter, Bennett, Embling, Hosegood, & Russell, 2016).

Our estimates of average movement rates are consistent with previous work. In cod, horizontal movement rates while in the migratory state are shown to be approximately 13.5 km/day which is comparable to past observations (Hobson et al., 2009) and laboratory studies (Bainbridge, 1957; Videler & Wardle, 1991). In plaice, previous research reports that seven tagged individuals swam on average 255 ± 60.2 km during prespawning migrations (Hunter, Metcalfe, & Reynolds, 2003). Assuming an average migration time of 2–4 weeks (as noted in Hunter et al., 2003), our estimates of horizontal movement rates between 13 and 20 km/day seem reasonable. Therefore, we are confident that our choice of state labels is biologically meaningful for the species in question.

Much work has considered the horizontal and vertical movements of Atlantic cod (Hobson et al., 2007, 2009) and European plaice (Hunter, Metcalfe, Arnold, et al., 2004; Hunter, Metcalfe, O'Brien, Arnold, and Reynolds, 2004), noting strong seasonal dependence in the movement patterns of individual fish. Here we add confidence to these findings by providing a mechanistic view of how fish switch between two movement modes during their annual cycle. In particular, we show that cod and plaice are more likely to occupy a resident state during the summer months (April–September in plaice; June–November in cod). These periods are dominated by low horizontal and vertical movement rates, therefore our findings support the hypothesis that both species spend their summer in a sedentary state with minimal activity levels (Metcalfe et al., 2006; Righton et al., 2010). Movement rates then ramp up during the winter and early spring (October–March in plaice; December–May in cod), resulting in a collective shift in state. As in previous studies (Hobson et al., 2007; Hunter et al., 2004b), we interpret this shift to be reflective of prespawning migrations, the onset of spawning and subsequent postspawning migrations. One limitation of the two‐state model considered here is that we cannot directly infer foraging or spawning behavior. Foraging and spawning events are likely to represent an immediate activity level, with both behaviors involving notable vertical displacement to and from the water column (Hobson et al., 2009). The inclusion of a third immediate state would be a relatively straightforward extension to model structure (see Vermard, Rivot, Mahévas, Marchal, & Gascuel, 2010; Peel & Good, 2011; Michelot et al., 2017 for examples of HMMs that consider >2 states). However, it is unlikely that the scale of these vertical excursions is large enough to allow classification at the daily time step. Therefore, we suggest that future studies either deploy more sophisticated tags which are capable of recording more refined information about the underlying movement process (e.g., accelerometers; Leos‐Barajas, Photopoulou, et al., 2017) or consider a nested hierarchical HMMs in which vertical and horizontal movements are recorded and classified at differing time scales (Leos‐Barajas, Gangloff, et al., 2017).

Over the last 70 years, landings data for the North Sea and English Channel demonstrate that catch per unit effort (CPUE) for demersal species is higher during the summer months (Righton, Townhill, & Van Der Kooij, 2009). Such increases in CPUE are undoubtedly linked to changes in the populations’ underlying movement behavior, as time spent on the seabed results in an increased vulnerability to commercial exploitation (Righton et al., 2009). By assuming that time spent in a resident state is linked to sea‐bottom dwelling, we show that cod and plaice aggregate in certain habitat types. For example, cod in the English Channel have greatest density in the deeper waters at the western mouth of the English Channel. In contrast, cod and plaice in the Southern North Sea aggregate in coastal waters off the English mainland. We also demonstrate that plaice in the German Bight remain exclusively within this region, suggesting the presence of a sedentary resident population in which fish spawn and forage in the same locality (previously noted in plaice by Hunter et al., 2004b and in cod by Neat et al., 2006). Such spatial information is essential for defining multispecies management measures, as strategies typically involve gear restrictions (Moustakas, Silvert, & Dimitromanolakis, 2006) aimed at limiting the exploitation of certain species/life stages and spatial fisheries closures aimed at protecting areas of particular importance for species survival, for example foraging and spawning grounds (Hunter et al., 2004b; Righton, Quayle, Hetherington, & Burt, 2007).

One limitation of our method is the way in which we deal with individual variation. Currently we assume that by analyzing the movements of a finite sample of data‐rich pathways (*n* = 34) we gain sufficient information about how the mean movement of each state is distributed throughout the population. We then expect the movements of all other individuals to be drawn from one of these distributions and make no attempt to explain any deviance away from this “expected” process. One way to improve our approach and make it more generic would be the inclusion of covariate information (Phillips, Patterson, Leroy, Pilling, & Nicol, 2015). For example, four Atlantic cod were unexpectedly classified solely to a resident state even though their movements occurred throughout the winter (November–April). *Post hoc* investigations reveal an average body length of ~56 cm which lies within the predicted range of length at first maturity (31–74 cm; Froese & Pauly, 2017). It is likely that immature fish act differently to their mature conspecifics (Sippel et al., 2015) and that tagging programmes like the one considered here include fish of differing sex and age (Carter et al., 2016). Consideration of these factors is beyond the scope of this paper. However, we believe that the inclusion of body length (see Towner et al., 2016 for an ecological example) or other individual covariates within the HMMs likelihood function would provide a fruitful avenue for future research.

Technological advancements in telemetry devices have led to huge efforts to track the movements of free‐roaming marine animals (Hays et al., 2016; Hussey et al., 2015). Tagging data are now seen as a valuable information source for stock assessment models (Sippel et al., 2015), monitoring the effectiveness of conservation efforts (e.g., McGowan et al., 2017; Raymond et al., 2015) and understanding population dynamics across vast spatial scales (Block et al., 2011; Hindell et al., 2016). However, there is no avoiding the fact that tags are expensive (McGowan et al., 2017), liable to occasional failure and often produce individual pathways that are of limited use (data‐poor or a low number of observations). Here, we have introduced a methodology that makes the process of scaling up inference about movement behaviors from individuals to population more readily achievable. Moreover, we illustrate how the adoption of our approach can make tagging studies more cost‐effective, as inference can still be gained from data‐poor movement paths without resorting to redeployment or a renewed effort to secure further funding.

## CONFLICT OF INTEREST

Authors declare no conflict of interests.

## AUTHORS’ CONTRIBUTIONS

C.A.G, T.A.P., and P.G.B. designed the methodology; C.A.G., P.G.B., J.L.B., and D.A.R. interpreted and analyzed the model's output; movement paths were derived and analyzed by D.A.R. and S.R.W.; C.A.G., J.L.B., P.G.B., D.A.R., and J.W.P. led the writing of the manuscript; C.A.G. and S.R.W. designed the figures. All authors contributed critically to the drafts and gave final approval for publication.

## DATA AND R CODE

The collated datasets for each fish species including estimated state sequences, geolocation estimates (latitude and longitude), and date stamps can be found on the CEFAS Data Hub (https://doi.org/10.14466/cefasdatahub.54). Example R code to run our HMM is included in Supplementary Information document 2 or can be downloaded from GitHub (https://github.com/cagriffiths1/Fish_HMM).
