# Supplementary material for: Scaling marine fish movement behavior from individuals to populations
Source: Ecol Evol. 2018 Jun 25;8(14):7031–43. doi: 10.1002/ece3.4223 (PMC6065275; doi:10.1002/ece3.4223)
Supplement: Supplementary file 1 [file ECE3-8-7031-s001.pdf]

Supplementary Information 1

Table S1. Summary of movement paths by sub-stock.

			**Movement path duration (number of days)**		
**Species**	**Sub-stock**	**Number of individuals**	**Minimum**	**Mean**	**Maximum**
**Atlantic cod** (*Gadus morhua*)	Southern North Sea	23	40	97	295
	English Channel	23	41	145	364
**European plaice** (*Pleuronectes platessa*)	Southern North Sea	24	42	205	399
	German Bight	10	56	183	356
	Central North Sea	27	49	131	368

Figure S1. HMM output for an individual movement path (duration = 295 days). Shown are the time-varying changes in state as the model switches between a resident (state 1) and a migrating (state 2) mode. The fish in question is an Atlantic cod from the Southern North Sea sub-stock, tagged on the 17th April 2001 and recaptured on the 5th February 2002. All horizontal (m) and vertical (m) movement rates have been log (natural log) transformed.

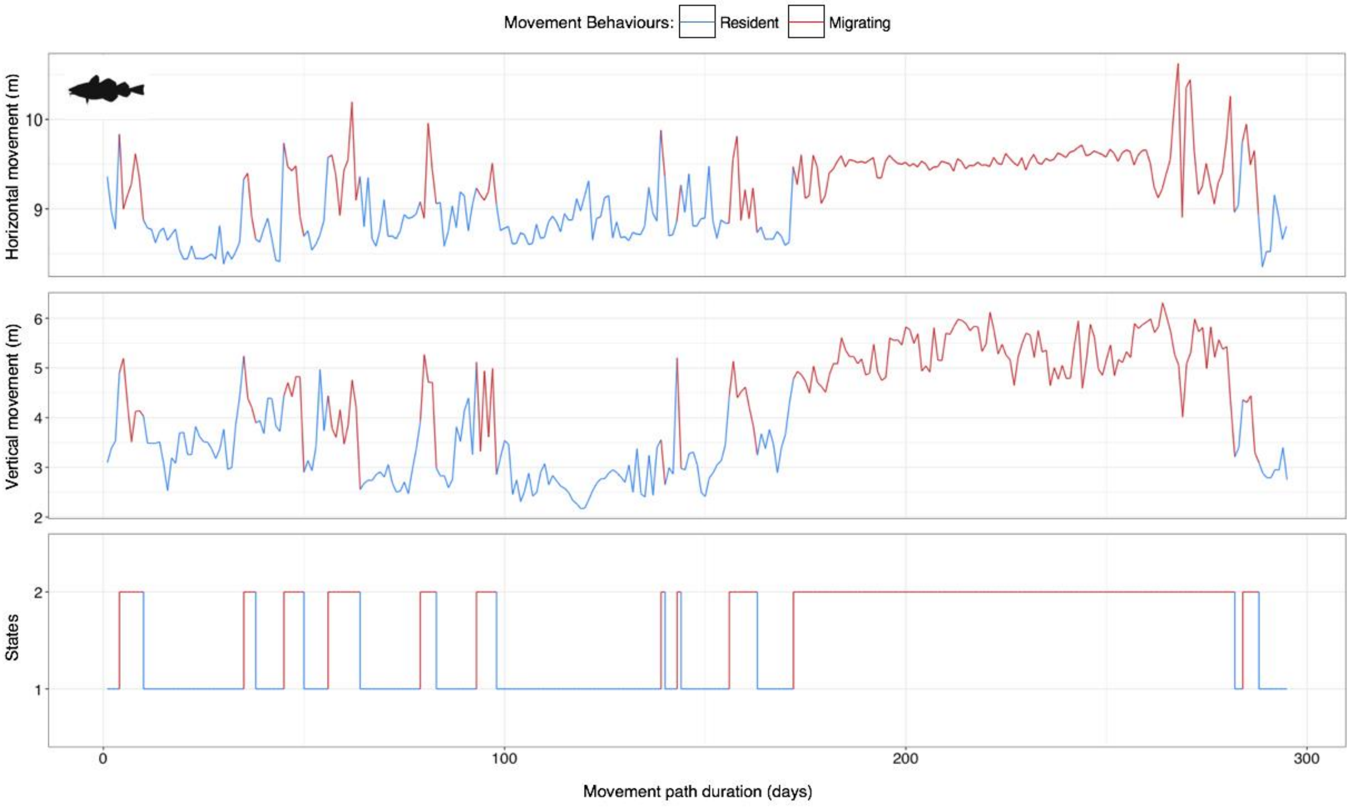

Figure S2. HMM output for an individual movement path (duration = 192 days). Shown are the time-varying changes in state as the model switches between a resident (state 1) and a migrating (state 2) mode. The fish in question is a European plaice from the Southern North Sea sub-stock, tagged on the 18th December 1997 and recaptured on the 27th June 1998. All horizontal (m) and vertical (m) movement rates have been log (natural log) transformed.

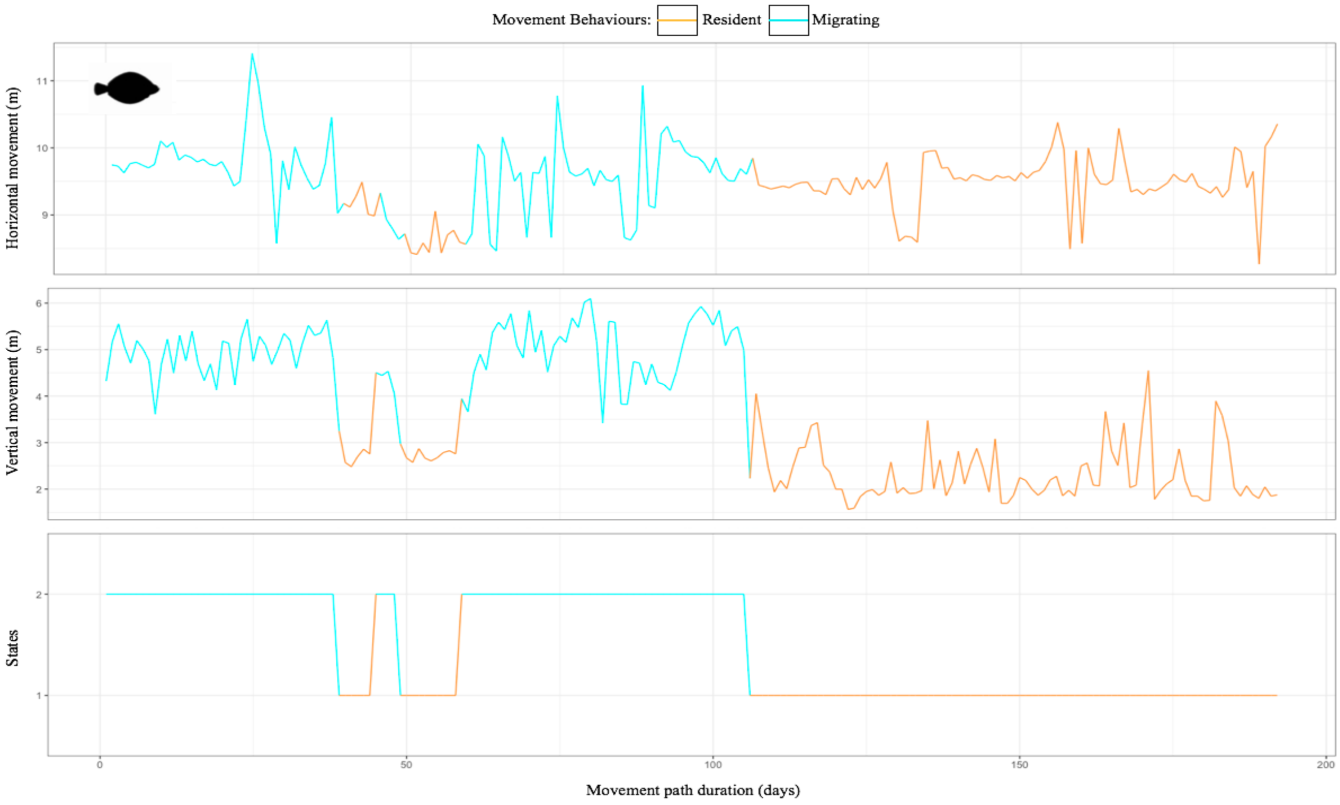

Figure S3. Criteria used to select individual movement paths based on published movement types. Selection is species-specific.

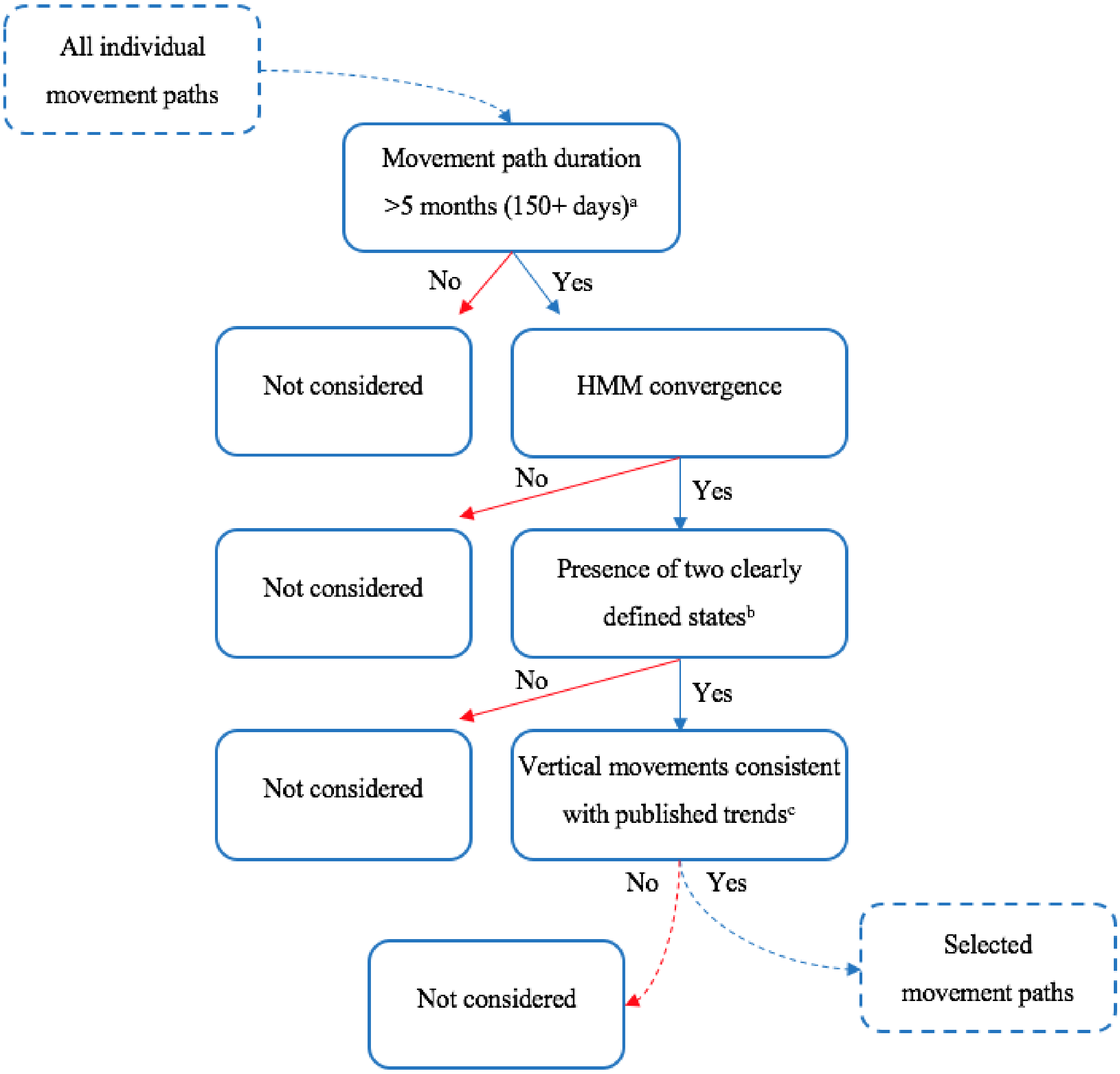

[a] So as to encompass any underlying seasonal shift in movement behaviour (Metcalfe, Hunter & Buckley 2006; Righton *et al.* 2010).

[b] One more active and one less active state (Metcalfe *et al.* 2006; Righton *et al.* 2010).

[c] For vertical movement types we refer to Hobson *et al.* (2007 - Atlantic cod) and Hunter *et al.* (2004a; b – European plaice).

Table S2. Summary of selected movement paths by sub-stock.

Species	Sub-stock	Number of individuals	Average duration of movement path (days)
**Atlantic cod** (*Gadus morhua*)	Southern North Sea	4	160
	English Channel	7	174
**European plaice** (*Pleuronectes platessa*)	Southern North Sea	10	241
	German Bight	7	201
	Central North Sea	6	171

Figure S4. Prior parameter distributions by state in Atlantic cod (n=11) and European plaice (n=23). Points and lines are movement path specific, points show the mean bivariate movement rate per state and ellipses show the highest density region sampled from each states covariance matrix. Plotted are all movement paths that were selected and synthesized into movement parameter priors following initial HMM runs. All horizontal (m) and vertical (m) movement rates have been log (natural log) transformed.

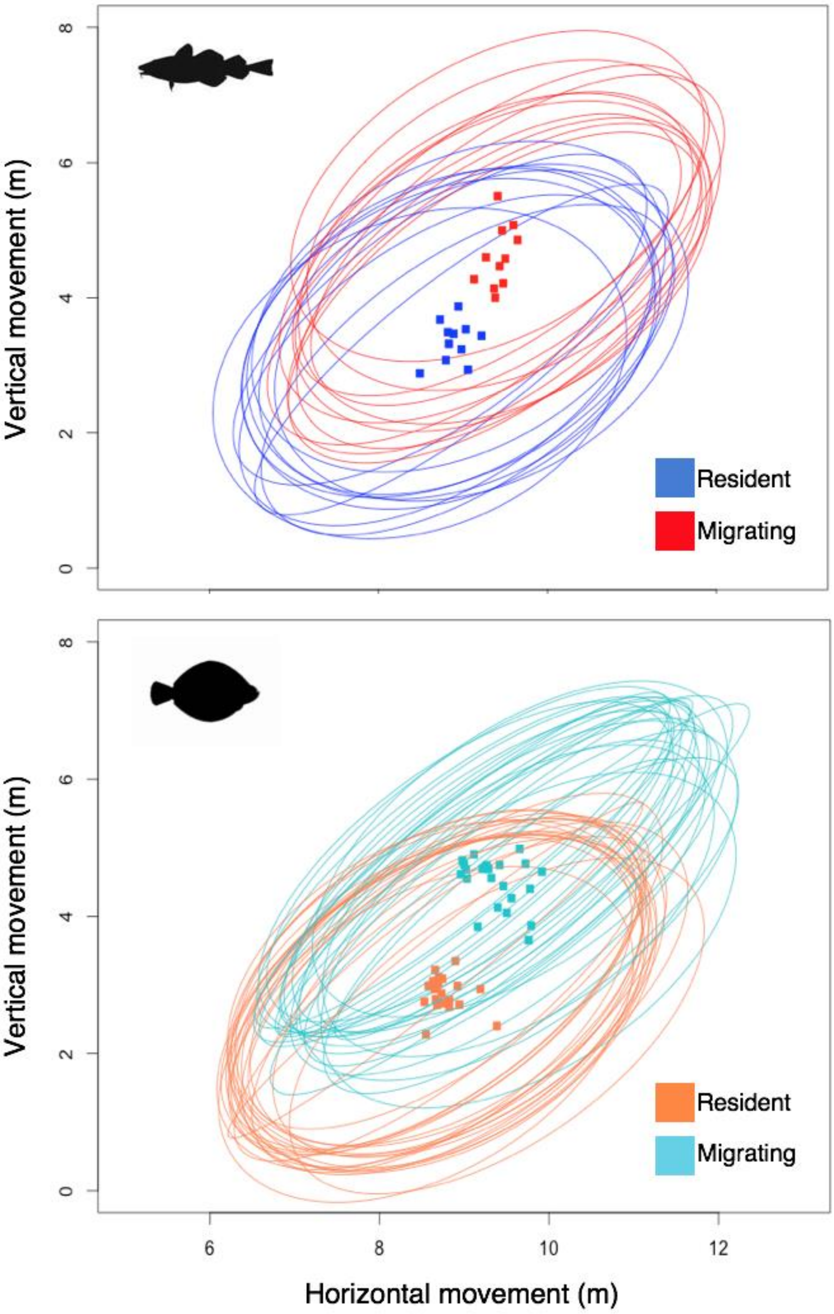

Figure S5. Movement parameter prior's influence on state classification. Shown are the results of running the same movement path through the HMM (top) and the adapted HMM (bottom) which includes prior information on each state's distribution. Each point is a bivariate movement rate and is plotted by state. Shown in purple is the migratory state and shown in cyan is the resident state, the mean movement rates within each state are plotted as crosses. Most notable is the re-classification of data points from a migratory state in the HMM to a resident state in the adapted HMM, which better reflects our prior knowledge about the movement behavior of demersal fish. The fish in question is a European plaice from the Southern North Sea and was tagged on the 3rd November 2004 (movement path duration = 349 days). All horizontal (m) and vertical (m) movement rates have been log (natural log) transformed.

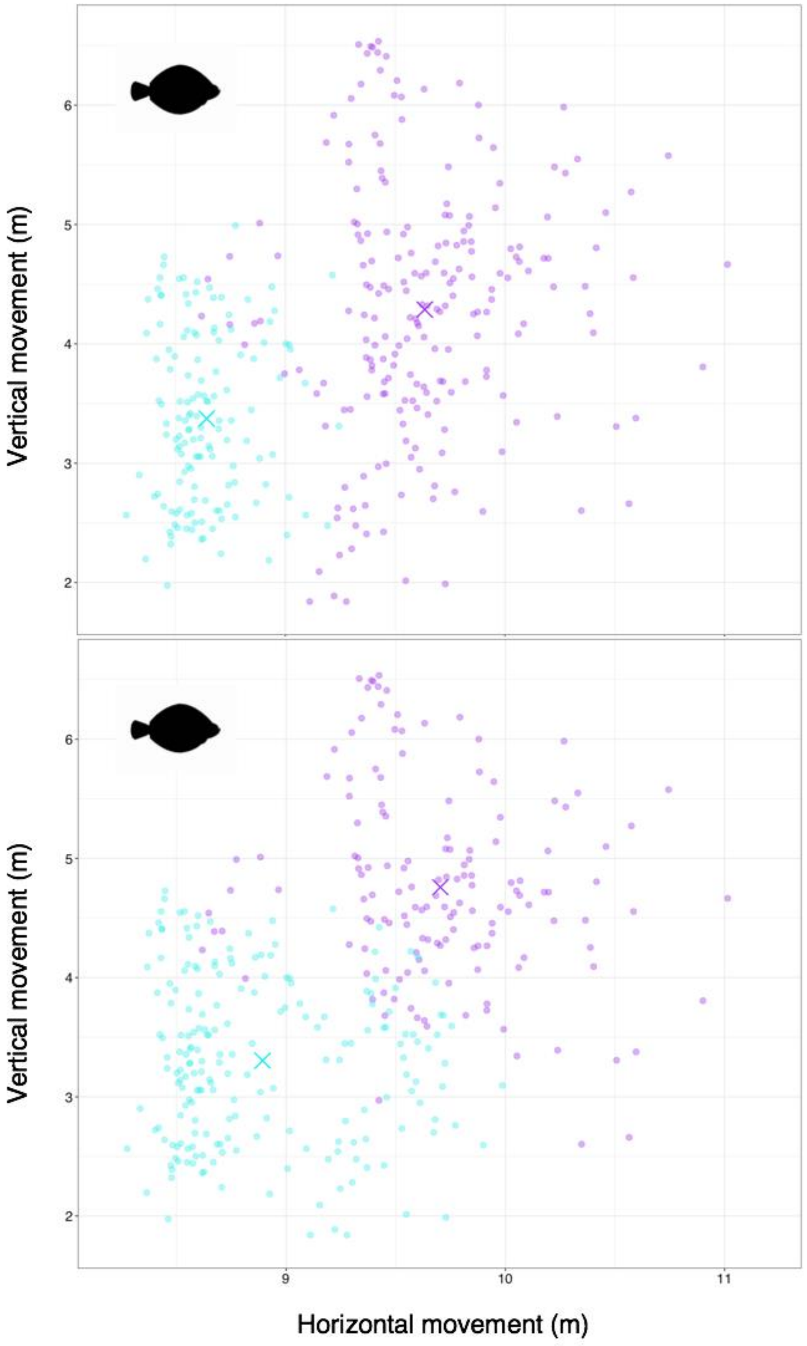

Figure S6. Mean bivariate movement rates by state in all 61 European plaice. All horizontal (m) and vertical (m) movement rates have been log (natural log) transformed.

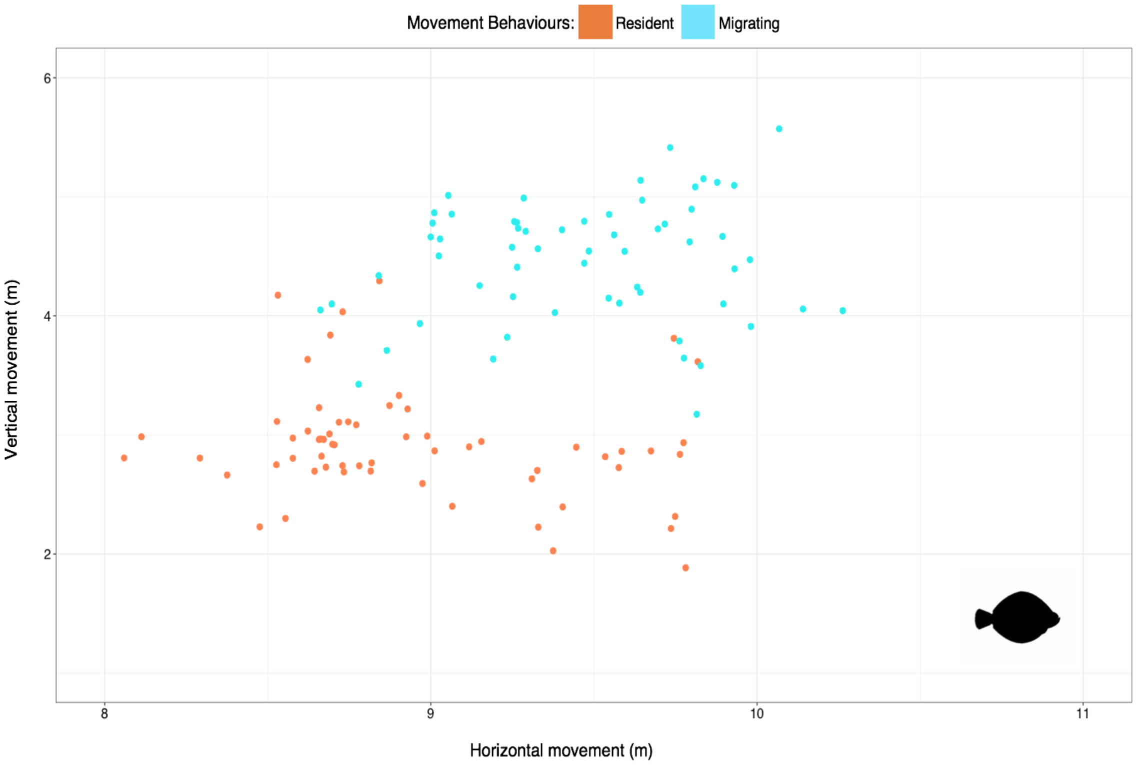

Figure S7. Influence of movement parameter prior on state classification process in an exceptionally data-poor movement path (Atlantic cod; path duration = 46 days). Shown are the results of running the same movement path through the HMM (top) and the adapted HHMM (bottom) which includes prior information on each state's distribution. Shown in blue and red are the resident and migrating state, respectively. The individual's mean movement rate per state (crosses) are plotted alongside the sub-stock's (English Channel) mean movement rate per state averaged across all 23 movement paths.

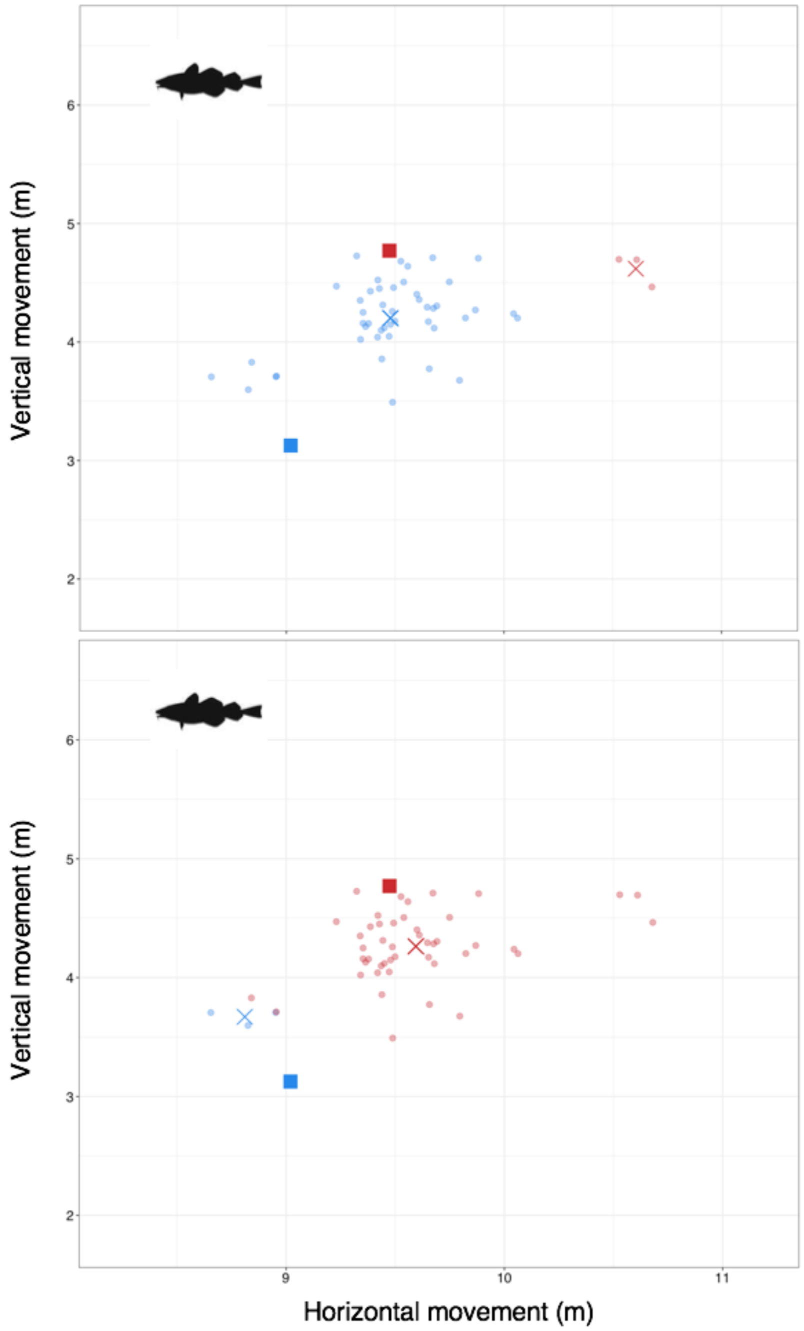

Table S3. Results of prior sensitivity analysis on HMM and adapted HMM state classification. Values are reported as the mean percentage change and maximum change in state across all tested individual movement paths (± 1 SD).

	**Prior**	**Sensitivity test**	**Mean change in state (%)**	**Maximum change in state (%)**
**Atlantic cod (*Gadus morhua*)**	Transition probability	1	1.83 (±2.61)	9.17
		2	1.27 (±2.52)	8.33
	Movement parameter	A	0.55 (±1.06)	3.23
		B	0.55 (±1.06)	3.37
**European plaice (*Pleuronectes platessa*)**	Transition probability	1	1.86 (±2.88)	13.59
		2	1.72 (±1.70)	5.63
	Movement parameter	A	2.27 (±2.23)	7.79
		B	2.45 (±2.49)	8.44

Figure S8. Predicted spatial utilisation distributions by state across all 5 sub-stocks of Atlantic cod (A) and European plaice (B). Plots are spilt into periods of resident dominant and migrating dominant, defined by a mean probability of observing a given state at a given time being > 0.5. All grid cells (5km^2) are illustrated in a colour gradient so as to illustrate the sum total number of days spent in a certain state in a given grid cell within a specified time period.

A

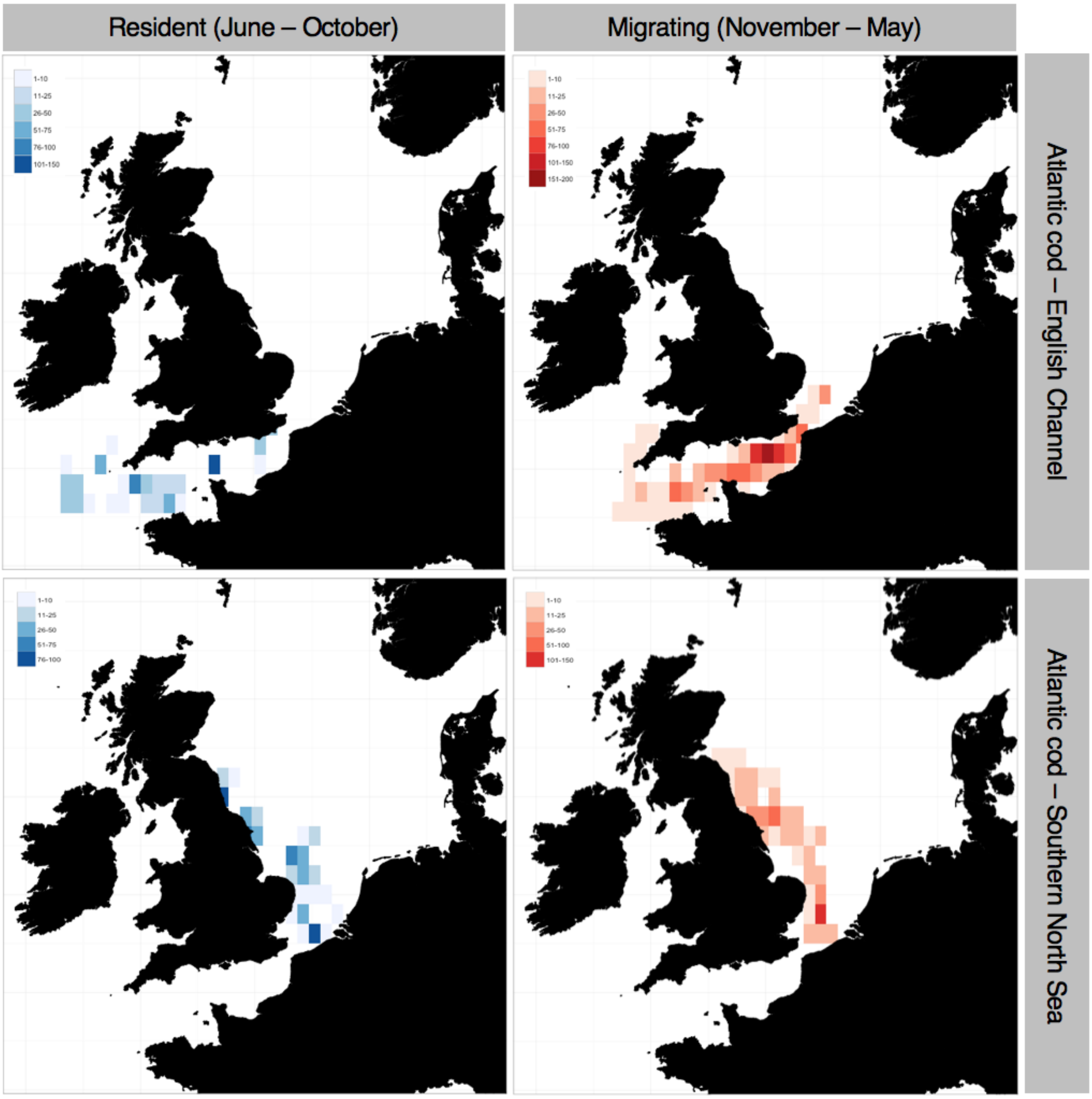

B

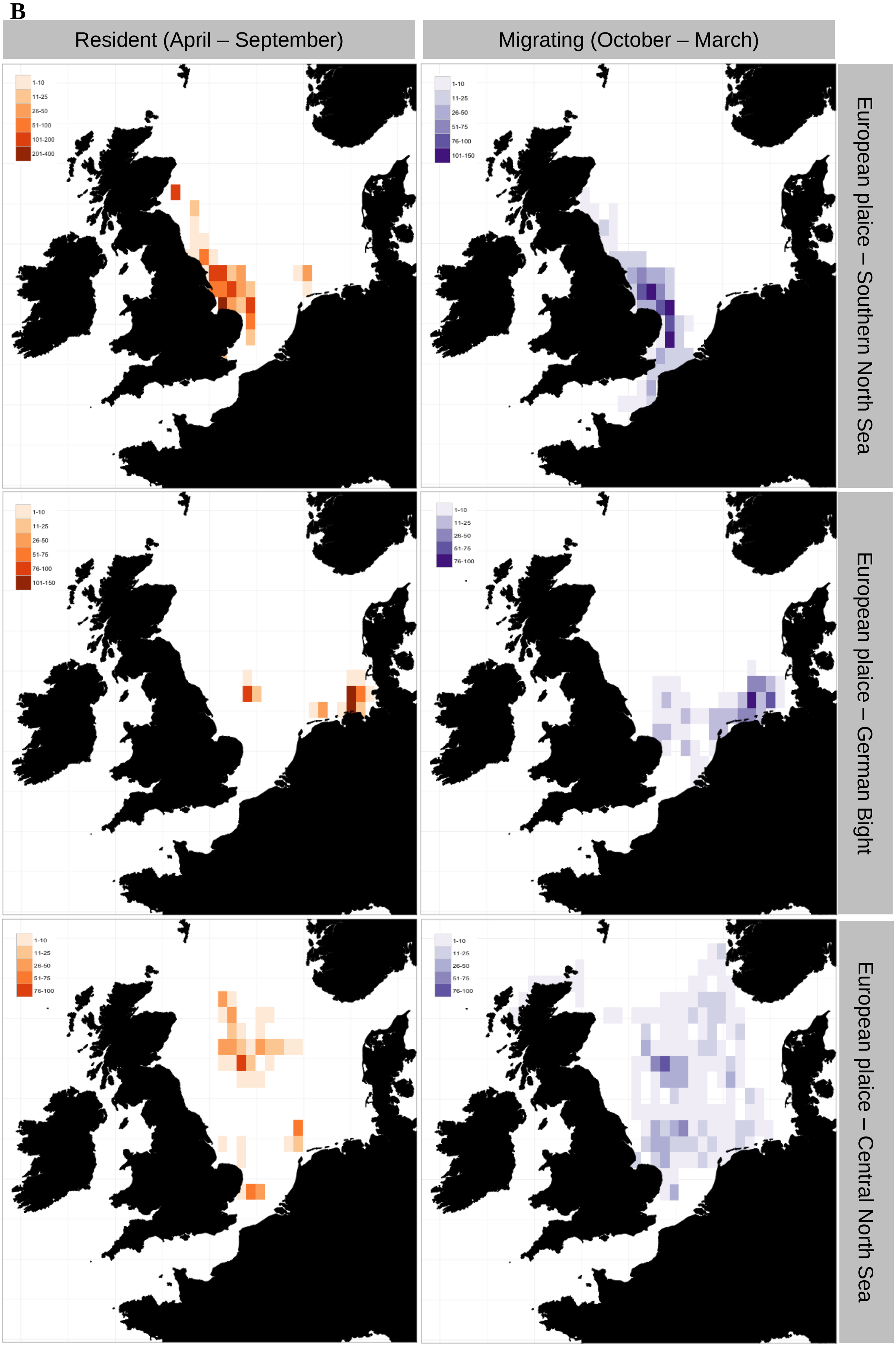
Resident (April – September)
Migrating (October – March)
European plaice – Southern North Sea
European plaice – German Bight
European plaice – Central North Sea
1-10
11-25
26-50
51-100
101-200
201-400
1-10
11-25
26-50
51-75
76-100
101-150
1-10
11-25
26-50
51-75
76-100
101-150
1-10
11-25
26-50
51-75
76-100
1-10
11-25
26-50
51-75
76-100
1-10
11-25
26-50
51-75
76-100

Figure S9. State dwell time distributions per state in Atlantic cod. The first and last period of time spent in a given state are omitted.

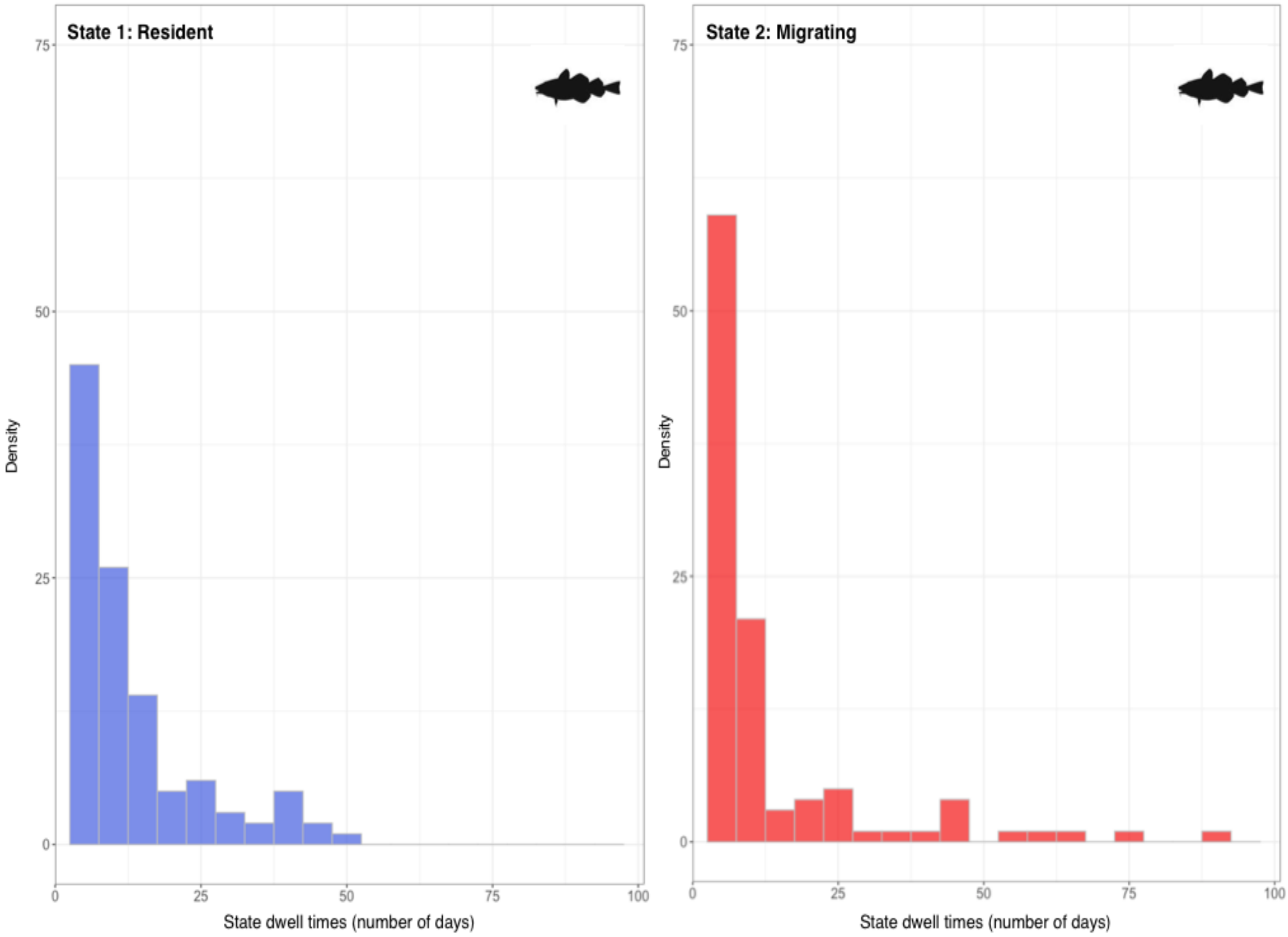

Figure S10. State dwell time distributions per state in European plaice. The first and last period of time spent in a given state are omitted.

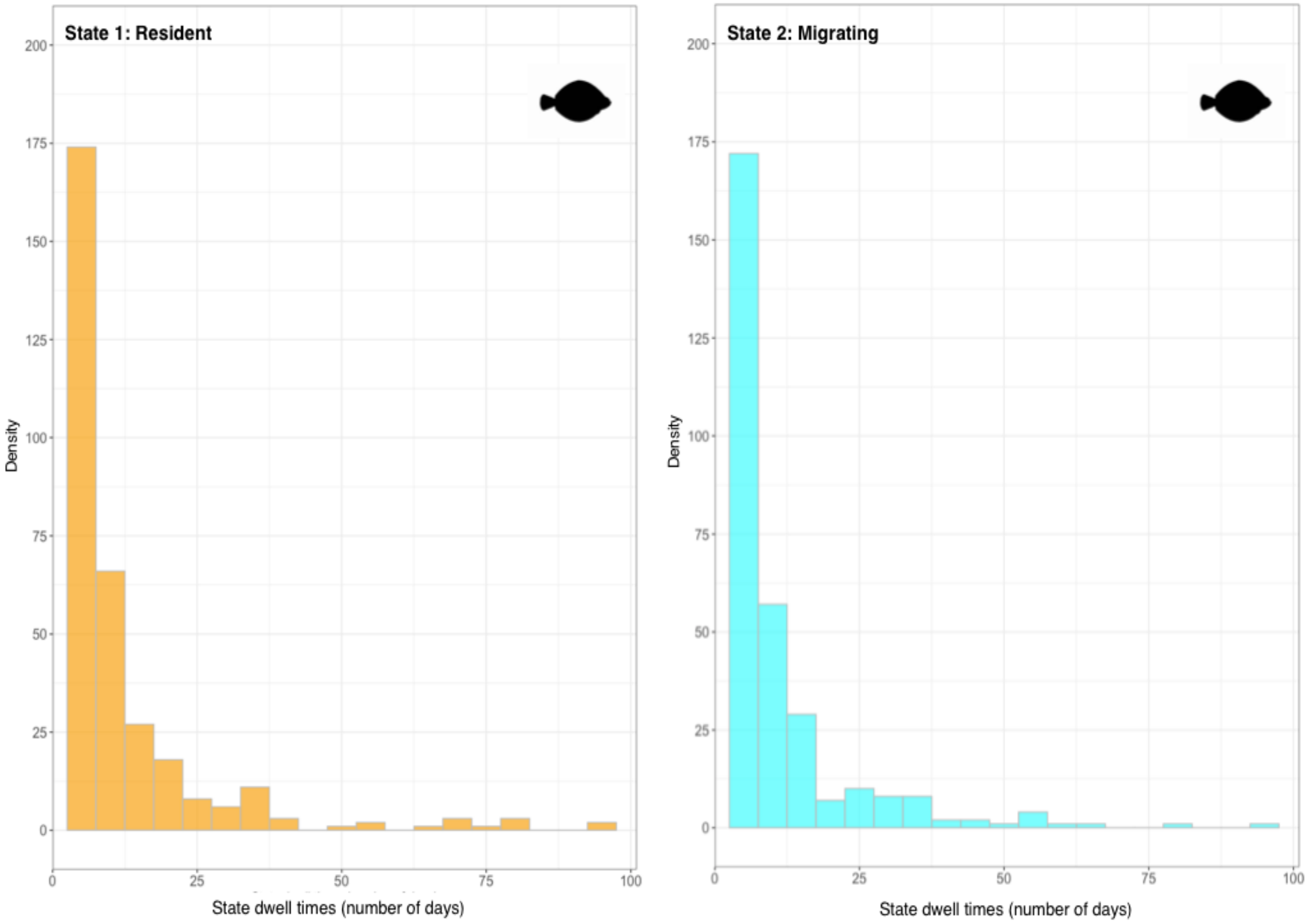

Figure S11. Observed vs. fitted values for a single Atlantic cod (number of observations = 115). Observed points (coloured) are plotted alongside the estimated mean of each state (black cross). Dashed ellipses are calculated from the estimated covariance and centred on the estimated mean of each state. All horizontal (m) and vertical (m) movement rates have been log (natural log) transformed.

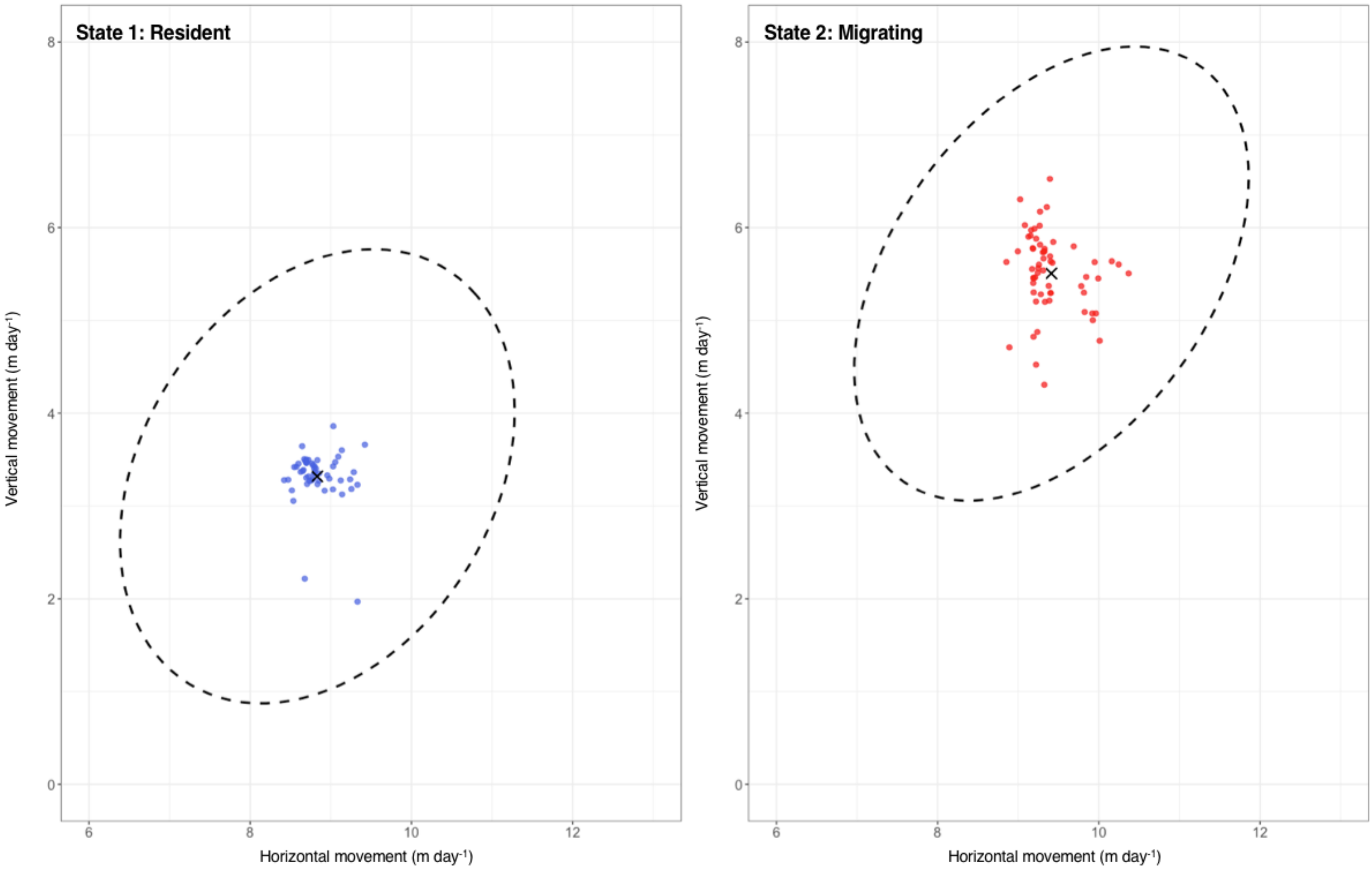

Figure S12. Observed vs. fitted values for a single European plaice (number of observations = 289). Observed points (coloured) are plotted alongside the estimated mean of each state (black cross). Dashed ellipses are calculated from the estimated covariance and centred on the estimated mean of each state. All horizontal (m) and vertical (m) movement rates have been log (natural log) transformed.

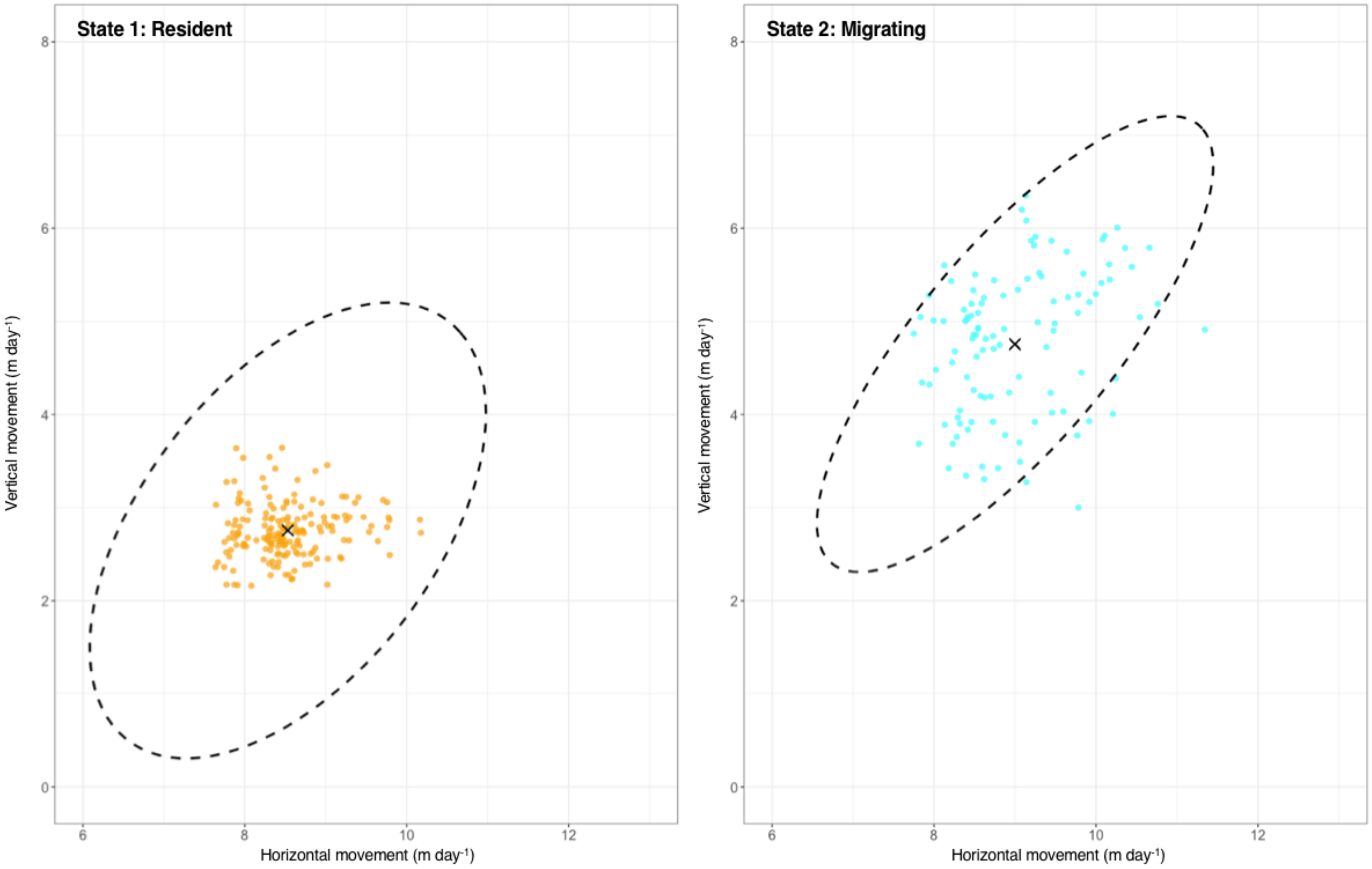

References

Hobson, V.J., Righton, D., Metcalfe, J.D. & Hays, G.C. (2007) Vertical movements of North Sea cod. *Marine Ecology Progress Series*, **347**, 101–110.

Hunter, E., Metcalfe, J.D., Arnold, G.P. & Reynolds, J.D. (2004a) Impacts of migratory behaviour on population structure in North Sea plaice. *Journal of Animal Ecology*, **73**, 377–385.

Hunter, E., Metcalfe, J.D., O'Brien, C.M., Arnold, G.P. & Reynolds, J.D. (2004b) Vertical activity patterns of free-swimming adult plaice in the southern North Sea. *Marine Ecology Progress Series*, **279**, 261–273.

Metcalfe, J.D., Hunter, E. & Buckley, A. (2006) The migratory behaviour of North Sea plaice: Currents, clocks and clues. *Marine and Freshwater Behaviour and Physiology*, **39**, 25–36.

Righton, D.A., Andersen, K.H., Neat, F., Thorsteinsson, V., Steingrund, P., Svedäng, H., Michalsen, K., Hinrichsen, H.H., Bendall, V., Neuenfeldt, S., Wright, P., Jonsson, P., Huse, G., Van Der Kooij, J., Mosegaard, H., Hüssy, K. & Metcalfe, J. (2010) Thermal niche of Atlantic cod Gadus morhua: Limits, tolerance and optima. *Marine Ecology Progress Series*, **420**, 1–13.
