# Supplementary material for: Scaling marine fish movement behavior from individuals to populations
Source: Ecol Evol. 2018 Jun 25;8(14):7031–43. doi: 10.1002/ece3.4223 (PMC6065275; doi:10.1002/ece3.4223)
Supplement: Supplementary file 2 [file ECE3-8-7031-s002.pdf]

```
################################################################################
# Example HMM code.
################################################################################
#
#AUTHORS:
#----------------
#Christopher Griffiths (cagriffiths1@sheffield.ac.uk)
#Toby Patterson (Toby.Patterson@csiro.au)
#Paul Blackwell (p.blackwell@sheffield.ac.uk)
#
#NOTES:
#---------------
#R (www.r-project.org; R version 3.4.0) code to fit a 2-state hidden Markov model (HMM) to an example bivariate movement path. The maximum likelihood estimation, filtering and smoothing all assume that movements are distributed according to a Gaussian distribution. Both horizontal and vertical movements are measured in metres per day and are log transformed as in Griffiths et al. (2018). The code is more-or-less hard-wired for a 2-state model, identifying one less active state and one more active state, although the inclusion of more states is straightforward. As in Griffiths et al. (2018) the two states are labeled as resident (low horizontal and vertical movements) and migrating (high horizontal and vertical movements).
#
#The example movement path is recorded from an Atlantic cod (Tag ID: 1186) that was tagged on the 25th March 2005 in the central English Channel and re-captured on the 18th January 2006. The fish undertakes intermittent phases of migration and resident behaviour immediately after release, before shifting to the migratory state as it moves in a south-westerly direction through the Channel. It then switches state and spends over 197 days consecutively (June-November) in the resident state within the deeper waters of the Celtic Sea. In early December a second major shift in behaviour occurs, as the fish moves back into the more active migratory state and moves directly through the Channel and into the Southern North Sea. This fish’s horizontal movements are also analysed in Pedersen et al. 2008 (Canadian Journal of Fisheries and Aquatic Sciences 65:2367–2377).
#
#Currently a single prior is acting on the state classification process, the transition probability prior which penalises the likelihood of state transition to an order of 100 days, as in Griffiths et al. (2017). The transition probability prior can easily be adapted to reflect what is known about the species in question. Example code to include 4 Gaussian movement priors on each states distribution, 2 per state, is also provided in lines 90-98. These are not needed in this case due to the presence of two clearly defined movement behaviours however as in Griffiths et al. (2018) we would recommend implementing these when applying HMMs to a large dataset of highly variable movement paths.
#
#
#COPYRIGHT:
#---------------
#This code is provided without warranty or any guarantees of any kind. It is free to distribute but please acknowledge the authors and reference appropriately.
#

################################################################################
# Required packages.
################################################################################

# Step 1. Install all required packages.
required.packages <- c("R.matlab","RgoogleMaps","marmap","maps","mapdata","gplots","zoo",
                       "ggplot2","reshape2","scales","plyr","matrixStats", "mvtnorm", "ellipse")
new.packages <- required.packages[!(required.packages %in% installed.packages()[,"Package"])]
if(length(new.packages)>=1){lapply(new.packages, install.packages)}
```

```
lapply(required.packages, require, character.only=T) #Should produce 14 'TRUE" statements.

##################################################################################
# Functions.
##################################################################################

# Step 2. Run all 7 functions
#This function finds the logit of one or more values – turns real values in probabilities, between [0,1].
logit <- function(p) log(p / (1-p))

#This function finds the inverse logit of one or more values – turns probabilities [0,1] into real values.
inv.logit <- function(X) exp(X) / (1+exp(X))

#Make a 2-state transition matrix.
make.tmat <- function(p, m) {
  tmat <- matrix(0, m , m)
  tmat[1,1] <- p[1]
  tmat[1,2] <- 1 - tmat[1,1]
  tmat[2,2] <- p[2]
  tmat[2,1] <- 1 - tmat[2,2]
  tmat
}

#Compute the negative log likelihood & smoothed state probabilities for a 2-state bivariate hidden
 Markov model - for model details see Griffiths et al. (2018).
hmm.like <- function (par,y,m=2,smootho=FALSE ) {
  p <- par # stupid way to get around optim.
  p.tmat <- inv.logit(p[1:m]) # transition matrix parameters.
  mu.1 <- p[(m+1):(m+2)]  # means state 1.
  sig.1 <- exp(p[(m+3) : (m+4)]) # 2 covariance params (state 1).
  rho.1 <- (exp(p[m+5])-1)/(exp(p[m+5])+1) # state 1 correlation coefficient.
  mu.2 <- p[(m+6):(m+7)]  # means state 2.
  sig.2 <- exp(p[(m+8):(m+9)]) # 2 covariance params (state 2).
  rho.2 <- (exp(p[m+10])-1)/(exp(p[m+10])+1) # state 2 correlation coefficient.

  tmat <- make.tmat(p.tmat, m) #create transition matrix.
  mu <- rbind(mu.1, mu.2)
  sig <- array(0, c(m,m,m))
  sig[,,1] <- matrix(c(sig.1[1]^2, rho.1*sig.1[1]*sig.1[2],
                     rho.1*sig.1[1]*sig.1[2],sig.1[2]^2), 2,2)
  sig[,,2] <- matrix(c(sig.2[1]^2, rho.2*sig.2[1]*sig.2[2],
                     rho.2*sig.2[1]*sig.2[2],sig.2[2]^2), 2,2)
  nt <- nrow(y)

  #Pre-compute data likelihoods.
  dlik <- matrix(0,m,nt)
  for(i in 1:m) { # loop over possible states.
    dlik[i,] <- dmvnorm(y,mu[i,], sig[,,i])
  }
  nt <- nrow(y)
  s.p <- s <- matrix(0, m,nt)
  s[,1] <- rep(1/m, m) #Likelihood of the being state 1 or 2 at time point 0.
  LL <- 0.0

  ### Transition probability prior ###
```

```
  LL <- LL + dbeta(tmat[1,1], 99, 1, log = TRUE) + dbeta(tmat[2,2], 99, 1, log=TRUE)

  ### Movement parameter prior - Examples of how state dependent priors can be included.
  # Expected mean & SD of horizontal movement in state 1 #
  ### LL <- LL + dnorm(mu[1,1],mean=...,sd=...,log=TRUE) ###
  # Expected mean & SD of vertical movement in state 1 #
  ### LL <- LL + dnorm(mu[1,2],mean=...,sd=...,log=TRUE) ###
  # Expected mean & SD of horizontal movement in state 2 #
  ### LL <- LL + dnorm(mu[2,1],mean=...,sd=...,log=TRUE) ###
  # Expected mean & SD of vertical movement in state 2 #
  ### LL <- LL + dnorm(mu[2,2],mean=...,sd=...,log=TRUE) ###

  #Forward recursive algorithm.
  for(i.t in 2:nt)  {
    s.p[,i.t] <- (tmat %*% s[,i.t-1])
    p.s <-  s.p[,i.t]* dlik[,i.t]
    vt <- sum(p.s)
    LL <- LL + log(vt + 1e-20)
    s[,i.t] <- p.s / vt
  }

  #Backwards smoothing loop.
    if(smootho)
  {
    for(i in (nt-1):1)
      s[,i] <- s[,i] * (  (s[,i+1] / s.p[,i+1]) %*% tmat )
    return(list(s=s, ml.state = apply(s, 2L, which.max)))
  }
  else {
    return(-1.0 * LL) #return negative log likelihood.
  }
}

#Model fitting/optimisation of parameter space – Maximum likelihood estimation.
fit.hmm <- function(y, p, ...) {
  #fit the model.
  fitto <- optim(par = p , fn = hmm.like, y=y, ...)
  est.par <- fitto$par
  #state prediction.
  state.pred <- hmm.like(p = est.par, y=y, smootho=TRUE)
  attr(state.pred, "params") <- est.par
  return(state.pred)
}

#Calculate initial model parameters.
int.params <- function (d) {
  cl <- kmeans(d, 2) #Simple K-means parameter estimation.
  means <- cl$centers
  clusters <- cl$cluster
  est <- data.frame(d, clusters)

  means_diff <-means[1,1]-means[2,1]
  if(means_diff > 0){
    mu.1 <- means[2,]
    mu.2 <- means[1,]
```

```
  }else{
    mu.1 <- means[1,]
    mu.2 <- means[2,]
  }
  if(means_diff > 0){
    est.state.2 <- subset(est, est$clusters == 2)
    est.state.1 <- subset(est, est$clusters == 1)
  }else{
    est.state.1 <- subset(est, est$clusters == 1)
    est.state.2 <- subset(est, est$clusters == 2)
  }

  est.state.1 <- data.frame(est.state.1[,1], est.state.1[,2])
  est.state.2 <- data.frame(est.state.2[,1], est.state.2[,2])
  est.cov.1 <- cov(est.state.1)
  est.cov.2 <- cov(est.state.2)

  p.tmat <- logit(c(0.95,0.95))
  sig.1 <- log(c(est.cov.1[1,1], est.cov.1[2,2]))
  sig.2 <- log(c(est.cov.2[1,1], est.cov.2[2,2]))
  rho.1 <- est.cov.1[1,2]
  rho.2 <- est.cov.2[1,2]

  #Sort initial parameter estimates into a single array.
  param.est <- c(p.tmat, mu.1, sig.1, rho.1, mu.2, sig.2, rho.2)
  return(param.est)
}

#Example plots.
example.plots <- function (data) {
  data$time <- seq(1, NROW(data), 1)
  data$state_seq <- as.factor(data$state_seq)
  data$lat <- dat$lat
  data$lon <- dat$lon

  # State dependent horizontal movement through time.
  p1 <- ggplot(data=data, aes(x=time, y=h, col=state_seq, group=1)) +
    geom_line() +
    theme_bw()+
    xlab('\nTime (days)') +
    ylab('Horizontal movement (m)\n') +
    theme(text = element_text(size=12), legend.position = "top") +
    scale_color_manual(name = "Movement Behaviour",
                       labels = c("Resident","Migrating"),
                       values = c("dodgerblue2", "firebrick3"))
  print(p1)

  # State dependent vertical movement through time.
  p2 <- ggplot(data=data, aes(x=time, y=v, col=state_seq, group=1)) +
    geom_line() +
    theme_bw()+
    xlab('\nTime (days)') +
    ylab('Vertical movement (m)\n') +
    theme(text = element_text(size=12), legend.position = "top") +
    scale_color_manual(name = "Movement Behaviour",
```

```
                  labels = c("Resident","Migrating"),
                  values = c("dodgerblue2", "firebrick3"))
  print(p2)

  # Estimated behavioural states through time.
  p3 <- ggplot(data=data, aes(x=time, y=state_seq, col=state_seq, group=1)) +
    geom_step() +
    theme_bw()+
    xlab('\nTime (days)') +
    ylab('Behavioural state\n') +
    theme(text = element_text(size=12), legend.position = "top") +
    scale_color_manual(name = "Movement Behaviour",
                  labels = c("Resident","Migrating"),
                  values = c("dodgerblue2", "firebrick3"))
  print(p3)

  #Set maps x and y limits.
  xlim <- c(-10,10)
  ylim <- c(48,60)

  #Source base map.
  coast <- map_data("worldHires", xlim = xlim,ylim=ylim)
  coast.poly <- geom_polygon(data=coast, aes(x=long, y=lat, group=group), colour= "black",
 fill="black", lwd=0.2)
  coast.outline <- geom_path(data=coast, aes(x=long, y=lat, group=group), colour= "black", lwd=0.2)

  #Create base map
  base <- ggplot()+
    coast.poly+
    coast.outline+
    coord_quickmap(xlim,ylim)+
    xlab("\nLongitude")+
    ylab("\nLatitude\n")+
    theme_bw()

 #Add state dependent movement tracks to base map.
  p4 <- base+
    geom_point(data = subset(data, data$state_seq == 1), aes(x=lon, y=lat), col='dodgerblue2',
          shape=20, alpha=0.8, size=3)+
    geom_point(data = subset(data, data$state_seq == 2), aes(x=lon, y=lat), col='firebrick3',
          shape=20, alpha=0.8, size=3)+
    geom_point(data = data[1,], aes(x=lon, y=lat), col='darkgreen', shape=17, size=4)+
    geom_point(data = data[NROW(data),], aes(x=lon, y=lat), col='red3', shape=17, size=4)

  print(p4)
}

##############################################################################
# Source example movement path.
##############################################################################

#Download movement data from the CEFAS DataHub and save.
#Source data from: https://doi.org/10.14466/CefasDataHub.54
#First request the data by clicking on 'Download as CSV', then enter your email.
#You'll recieve an email containing the licence information and the download link.
```

```r
setwd(…) #Set your own working directory.
dat <- read.csv("Atlantic_cod_Griffiths_et_al.csv") #Read in the Atlantic cod data frame.
dat <- subset(dat, dat$tag_ID == "1186_11032005") #Extract an example movement path.

#Data manipulation - log horizontal and vertical movement.
d <- data.frame(log(dat$horizontal_move_m), log(dat$vertical_move_m))

################################################################################
# Run HMM.
################################################################################

#Run functions.
param.est <- int.params(d = d)
test.like <- hmm.like(par = param.est, y= d)
sim.fit <- fit.hmm(y= d, p = param.est)

#Collate model output - state sequence and estimated parameters.
state.prob <- t(sim.fit$s)
colnames(state.prob) <- c('Probability of staying in S1', 'Probability of staying in S2')
data.output <- data.frame(d, sim.fit$ml.state, state.prob)
colnames(data.output) <- c('h', 'v', 'state_seq', 'prob.state.1', 'prob.state.2')

output.params <- attr(sim.fit,"params")
output.params[1:2] <- inv.logit(c(output.params[1], output.params[2]))
output.params[5:6] <- exp(c(output.params[5], output.params[6]))
output.params[10:11] <- exp(c(output.params[10], output.params[11]))
output.params <- data.frame(t(output.params))
colnames(output.params) <- c('trans.prob.1', 'trans.prob.2', 'mean.h.state.1', 'mean.v.state.1',
 'covar.state.1a', 'covar.state.1b', 'corr.coef.1', 'mean.h.state.2', 'mean.v.state.2', 'covar.state.2a',
 'covar.state.2b', 'corr.coef.2')

################################################################################
# Explore model output.
################################################################################

#Feed 'data.output' into the plotting function and explore model output.
output.params #Estimated parameters.
head(data.output) #Estimated state sequences.
example.plots(data = data.output) #Example plots.
```
